# IGF2BP3 Orchestrates Metabolic Reprogramming via Stabilizing m6A-Modified PLK1 mRNA to Promote Hepatocellular Carcinoma Progression

**DOI:** 10.3390/ijms27209245

**Published:** 2026-10-18

**Authors:** Guohua Ji, Ruining Liu, Yuli Ding, Shuang Wu, Yuzhou Luo, Cize Gao, Xiaobo Cui

**Affiliations:** 1 Department of Medical Genetics, School of Basic Medical Sciences, Harbin Medical University, Harbin 150081, China; jiguohua@hrbmu.edu.cn (G.J.); liuruining912@163.com (R.L.); 15234050197@163.com (Y.D.); 2020020030@hrbmu.edu.cn (S.W.); luoyuzhou@hrbmu.edu.cn (Y.L.); 2 Key Laboratory of Preservation of Human Genetic Resources and Disease Control in China ( Harbin Medical University), Ministry of Education, Harbin 150081, China

**Keywords:** hepatocellular carcinoma, IGF2BP3, PLK1, glycolysis, m6A

## Abstract

Epigenetic dysregulation, particularly N6-methyladenosine (m6A) modification, drives hepatocellular carcinoma (HCC) progression, yet the oncogenic role of the m6A reader IGF2BP3 remains incompletely understood. This study aimed to elucidate the functional contributions and molecular mechanisms of IGF2BP3 in HCC. Multi-omics databases and tissue microarrays were employed to evaluate *IGF2BP3* and *PLK1* expression, clinicopathological associations, and prognostic value via Kaplan–Meier, Cox regression, and ROC analyses. Molecular interactions were probed by RIP, luciferase reporter, MeRIP, dot blot, and actinomycin D assays. In vitro gain/loss-of-function experiments measured glycolytic metabolites, extracellular acidification rate (ECAR), oxygen consumption rate (OCR), mitochondrial mass/charge, ROS, and key proteins of glycolysis and oxidative phosphorylation (OXPHOS). Both *IGF2BP3* and *PLK1* were significantly upregulated in HCC versus adjacent non-tumor tissues, correlating positively with advanced clinical stage, higher tumor grade, and poorer overall survival. In the TCGA-LIHC discovery cohort, each showed an independent prognostic association with unfavorable outcomes after adjustment for established clinical covariates. *PLK1* mRNA was validated to carry m6A modifications and directly bind IGF2BP3, an interaction abolished by cycloleucine. Functionally, IGF2BP3 upregulated m6A-modified *PLK1* by enhancing its mRNA stability, leading to elevated HK2 expression, increased glucose-6-phosphate and lactate levels, and enhanced ECAR, while suppressing mitochondrial respiratory chain complexes, ROS production, and OCR. In conclusion, *IGF2BP3* is overexpressed in HCC and tightly associated with aggressive clinicopathological features and adverse outcomes. Altogether, these findings suggest that IGF2BP3 may enhance the stability of the m6A-modified *PLK1* transcript, orchestrating a metabolic rewiring from oxidative phosphorylation to glycolysis, revealing a potential IGF2BP3–m6A–PLK1 axis driving HCC aggressiveness and offering potential therapeutic targets for precision intervention.

## 1. Introduction

Liver cancer ranks as the third leading cause of cancer-related death worldwide, with hepatocellular carcinoma (HCC) accounting for approximately 85–90% of cases. Despite extensive research, HCC carries a dismal five-year overall survival rate of approximately 18%, underscoring its exceptional lethality [1]. Despite extensive research into molecularly targeted therapies and the recent advent of immunotherapy, no regimen has materially improved overall survival, leaving the therapeutic landscape for HCC profoundly unsatisfactory. Consequently, unraveling the mechanisms driving hepatocarcinogenesis is imperative for the development of novel diagnostic markers and effective treatments [2].

Cancer has long been viewed primarily as a genetic disease driven by cumulative mutations; however, energy metabolic reprogramming is now established as an equally essential hallmark of malignancy. Mutations in cancer-related genes frequently converge on the regulation of metabolic enzymes, rendering metabolic rewiring a fundamental basis for tumor cell survival and proliferation [3]. Under normoxic conditions, normal cells predominantly generate energy via oxidative phosphorylation (OXPHOS)-the same organellar hub where electron transfer chain leakage generates the bulk of intracellular reactive oxygen species (ROS). At basal levels, ROS act as signaling mediators that foster proliferation; however, once their production overwhelms the antioxidant buffering capacity, oxidative stress ensues, compromising organellar integrity and culminating in cellular dysfunction or death [4,5]. In stark contrast, rapidly proliferating tumor cells exhibit markedly increased glucose uptake and preferentially utilize aerobic glycolysis, even in the presence of adequate oxygen—a phenomenon known as the Warburg effect [6]. This glucose-centered metabolic adaptation not only supplies glycolytic intermediates for biosynthetic processes but also facilitates lactate production, which acidifies the tumor microenvironment and confers enhanced invasive capacity. Such metabolic reprogramming is particularly pronounced during hepatocarcinogenesis, positioning it as a pivotal driver of HCC initiation and progression [7,8,9].

N6-methyladenosine (m6A) modification, the most prevalent internal epitranscriptomic mark in eukaryotic mRNAs, dynamically regulates various aspects of RNA metabolism, including translation, splicing, degradation, and nuclear export [10]. The biological fate of m6A-modified transcripts is largely dictated by “reader” proteins, which recognize specific m6A motifs and orchestrate subsequent RNA processing [11,12]. Insulin-like growth factor 2 mRNA-binding protein 3 (IGF2BP3) is a prominent member of the IGF2BP family of RNA-binding proteins. Functioning as a canonical m6A reader, IGF2BP3 specifically recognizes conserved m6A-modified sequences and, in an m6A-dependent manner, enhances the stability and translation of target oncogenic mRNAs under both physiological and stress conditions [13,14,15,16,17]. Accumulating evidence indicates that the dysregulation of IGF2BP3 exerts widespread oncogenic effects by stabilizing transcripts that drive tumor progression [15,18]. However, its precise role in orchestrating metabolic reprogramming in HCC remains not fully characterized.

Polo-like kinase 1 (PLK1) is a conserved serine/threonine kinase that orchestrates mitotic progression. It is frequently overexpressed across malignancies, and in HCC, its upregulation associated with advanced stage and poor prognosis [19]. Beyond this canonical role, PLK1 hyperactivation rewires cellular energetics by potentiating aerobic glycolysis while suppressing mitochondrial OXPHOS, thereby reinforcing the Warburg effect to support anabolic biomass production [20,21].

Given that PLK1 and IGF2BP3—an m6A-binding protein—are often co-upregulated in HCC, we reasoned that IGF2BP3 may couple epitranscriptomic regulation to metabolic reprogramming via post-transcriptional regulation of *PLK1* transcripts, shifting HCC metabolism toward aerobic glycolysis and driving tumor aggressiveness. Our study aims to identify a robust prognostic biomarker and expose a metabolic addiction that could be therapeutically exploited in HCC.

## 2. Results

### 2.1. IGF2BP3 Is Upregulated in HCC and Associated with Poor Prognosis

**Figure 1:**
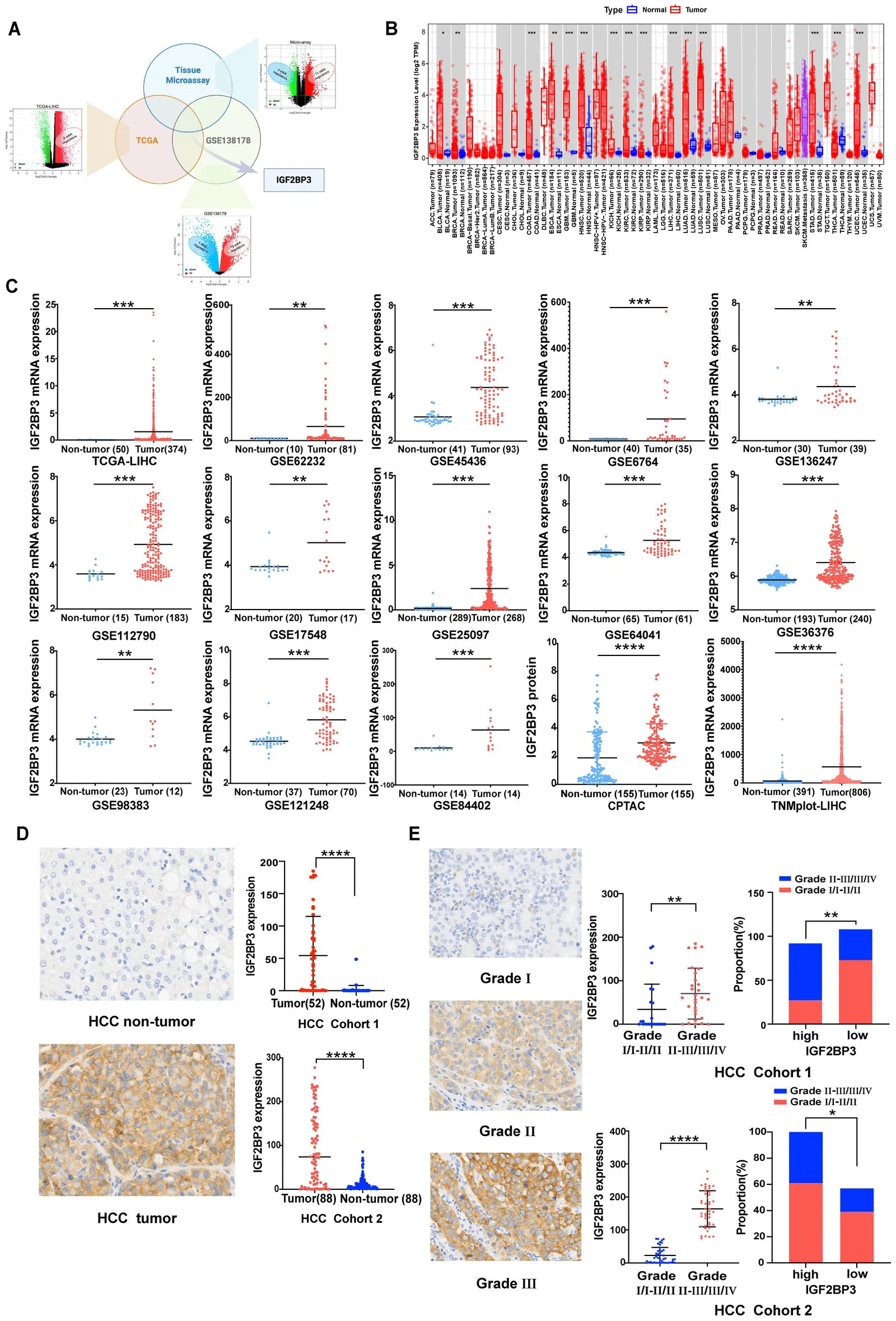

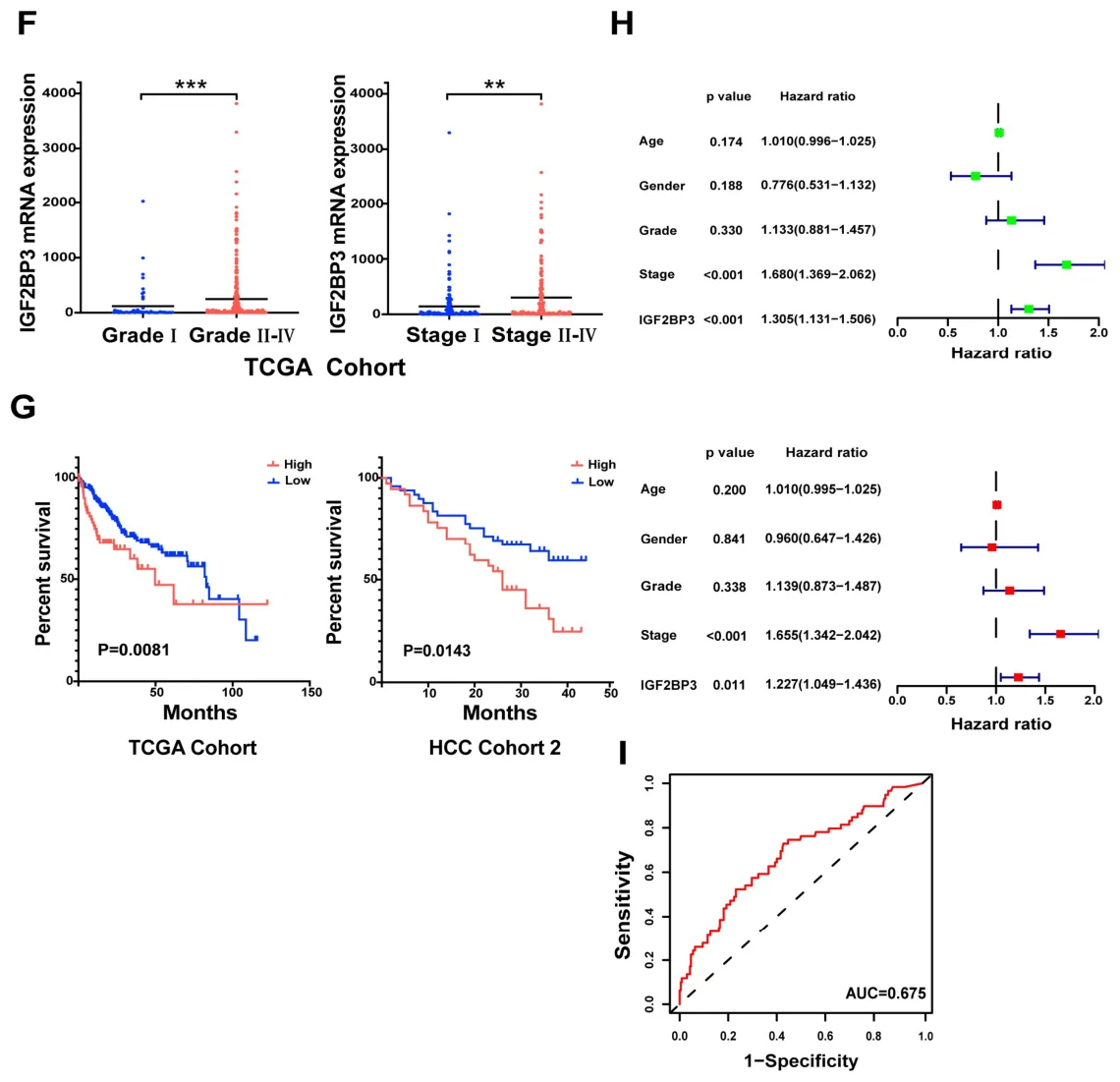
*IGF2BP3* is upregulated in hepatocellular carcinoma and associated with adverse prognosis. (**A**) Integrated screening of m6A regulatory genes from TCGA-LIHC, GSE138178, and tissue microarray data (|log_2_FC| ≥ 1, *p* < 0.05). In the Venn diagram, red, green and blue denote the TCGA-LIHC gene set, GSE138178 gene set and m6A epitranscriptomic microarray gene set, respectively. In volcano plots, red dots represent upregulated genes, while green dots (for TCGA-LIHC and m6A epitranscriptomic microarray datasets) or blue dots (for GSE138178 dataset) represent downregulated genes. (**B**) Pan-cancer expression landscape of *IGF2BP3* across various tumor types. Red indicates tumor samples and blue indicates normal samples. (**C**) Multi-database validation (TCGA-LIHC, GEO, CPTAC, TNMplot) of *IGF2BP3* upregulation at both mRNA and protein levels. (**D**) Representative immunohistochemistry (IHC) and quantitative analysis of IGF2BP3 in tumor versus adjacent non-tumor tissues from two independent cohorts (Cohort 1, *n* = 52; Cohort 2, *n* = 88). (**E**) IHC staining across pathological grades (I–III), with quantitative comparison between low grade (I–II) and high grade (III–IV) groups. (**F**) *IGF2BP3* expression stratified by pathological grade and clinical stage. (**G**) Kaplan–Meier curves for overall survival according to *IGF2BP3* expression. (**H**) Univariate and multivariate Cox regression analyses evaluating the prognostic association of *IGF2BP3* with overall survival. (**I**) ROC curve assessing the predictive performance of *IGF2BP3* expression level (* *p* < 0.05, ** *p* < 0.01, *** *p* < 0.001, **** *p* < 0.0001).

To identify key m6A regulators in HCC, we integrated transcriptomic (TCGA-LIHC, GSE138178) and epitranscriptomic (tissue microarray) data, using a cutoff of |log_2_FC| ≥ 1 and *p* < 0.05. *IGF2BP3* emerged as a significantly upregulated core regulator (Figure 1A). Pan-cancer profiling (Figure 1B) and multi-center validation (TCGA, GEO, CPTAC, TNMplot) consistently confirmed its overexpression at both mRNA and protein levels in tumors versus adjacent normals (Figure 1C). In two independent TMA cohorts, IGF2BP3 protein was elevated in tumors relative to paired normals, associated positively with grade, and was more frequent in advanced grades (Figure 1D,E), supporting its oncogenic potential. High *IGF2BP3* expression also associated with advanced stage/grade (Figure 1F) and shorter overall survival (Figure 1G). Univariate Cox regression identified elevated *IGF2BP3* expression as a significant factor associated with poorer overall survival. Multivariate analysis adjusted for age, sex, grade and stage revealed an independent prognostic association between upregulation and adverse outcomes (HR = 1.227, 95% CI: 1.049–1.436, *p* = 0.011). ROC curves validated its predictive accuracy (Figure 1H,I).

### 2.2. IGF2BP3 Drives Metabolic Reprogramming in HCC

**Figure 2:**
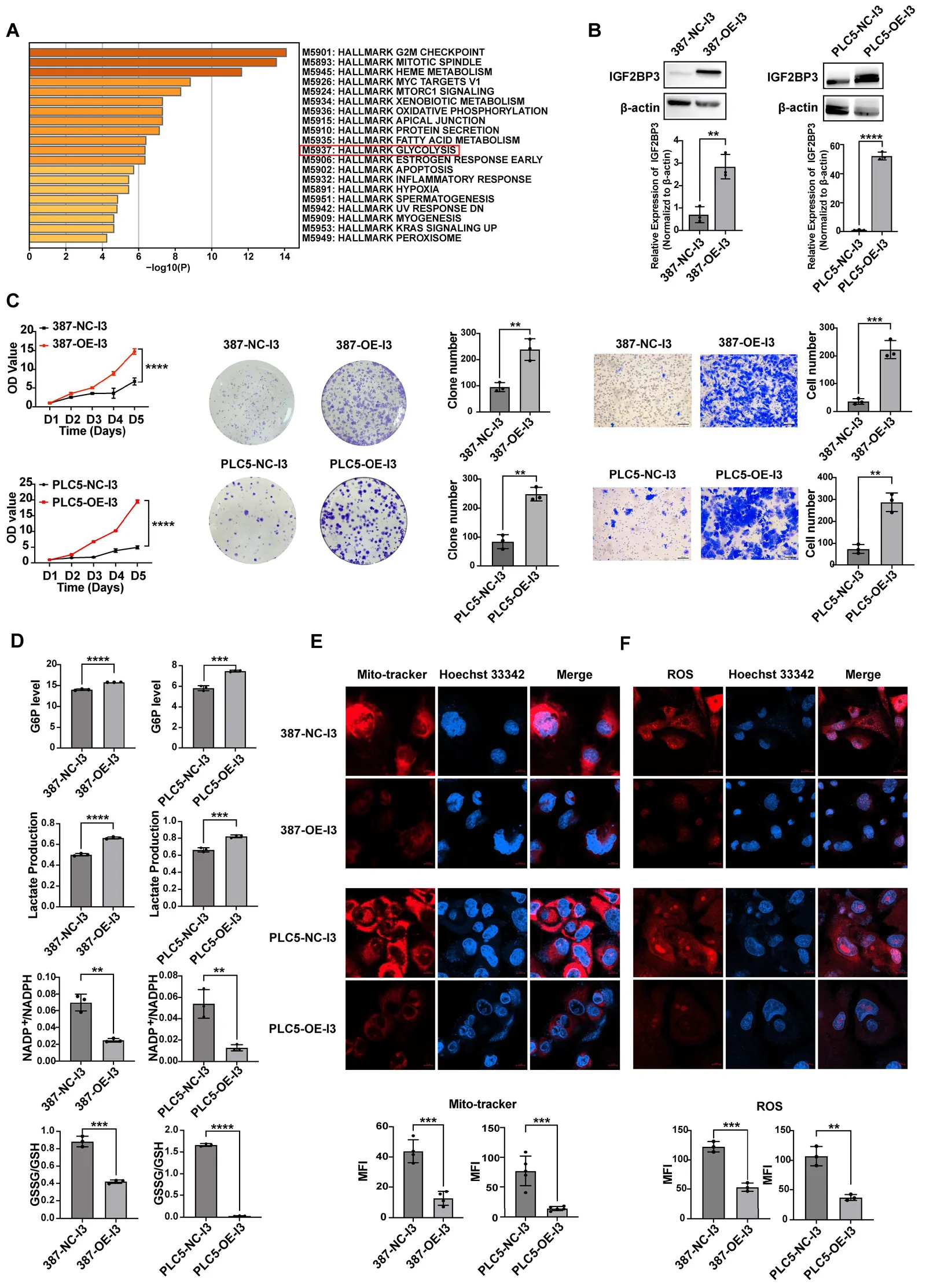

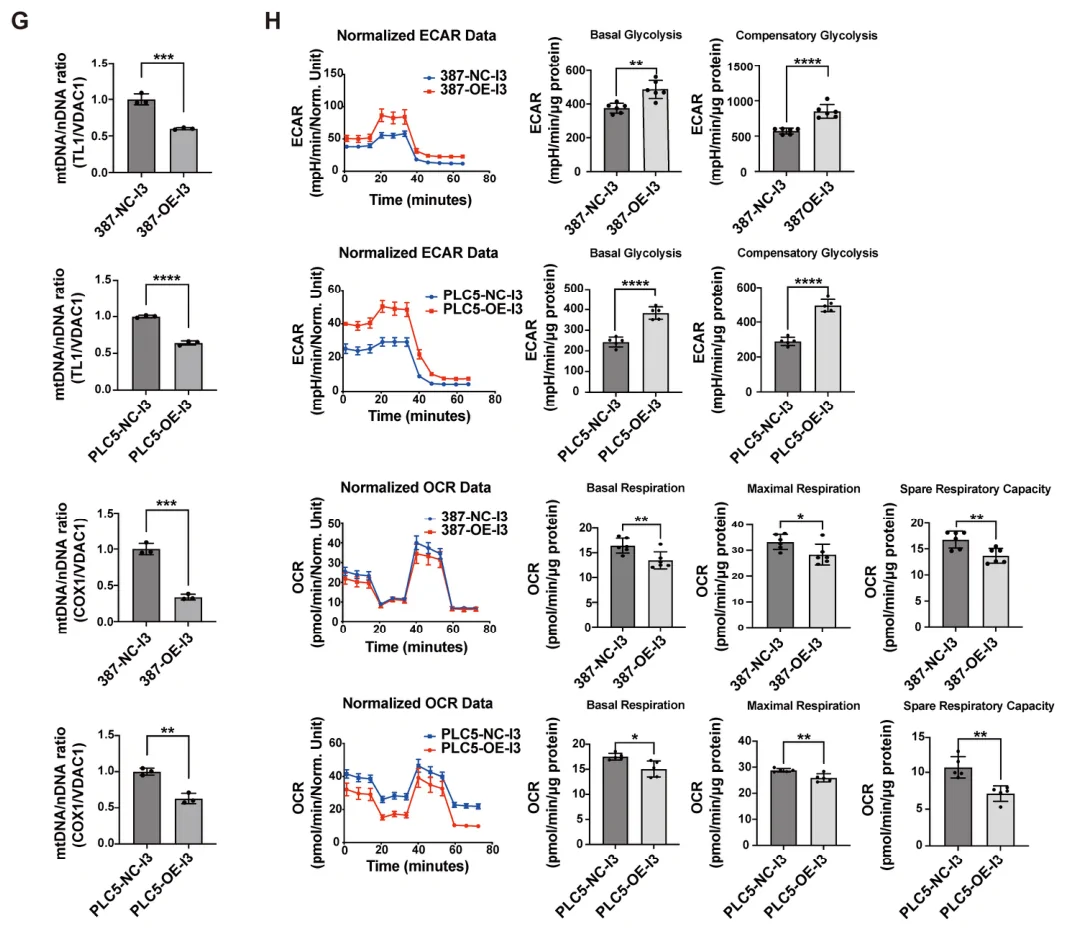

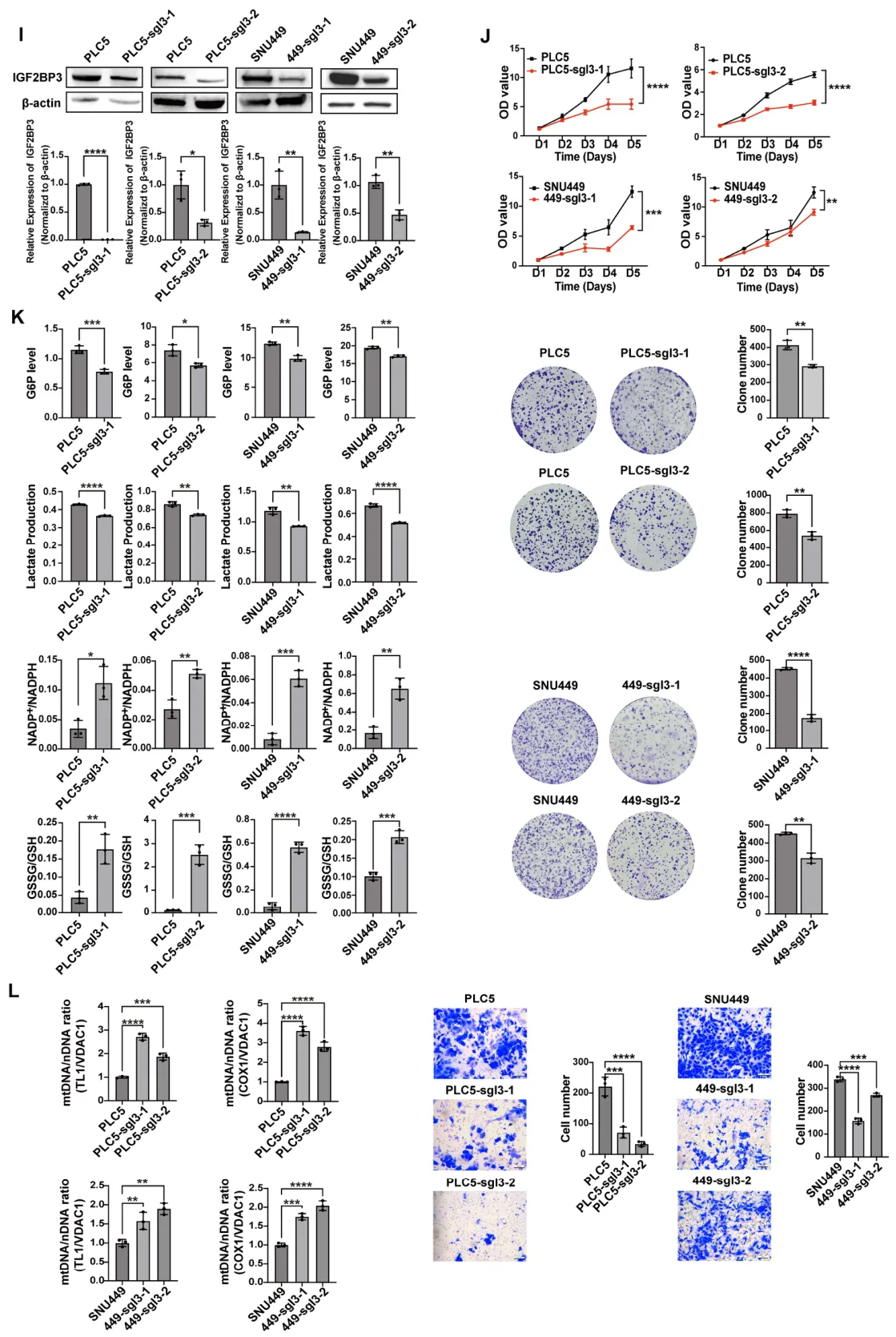

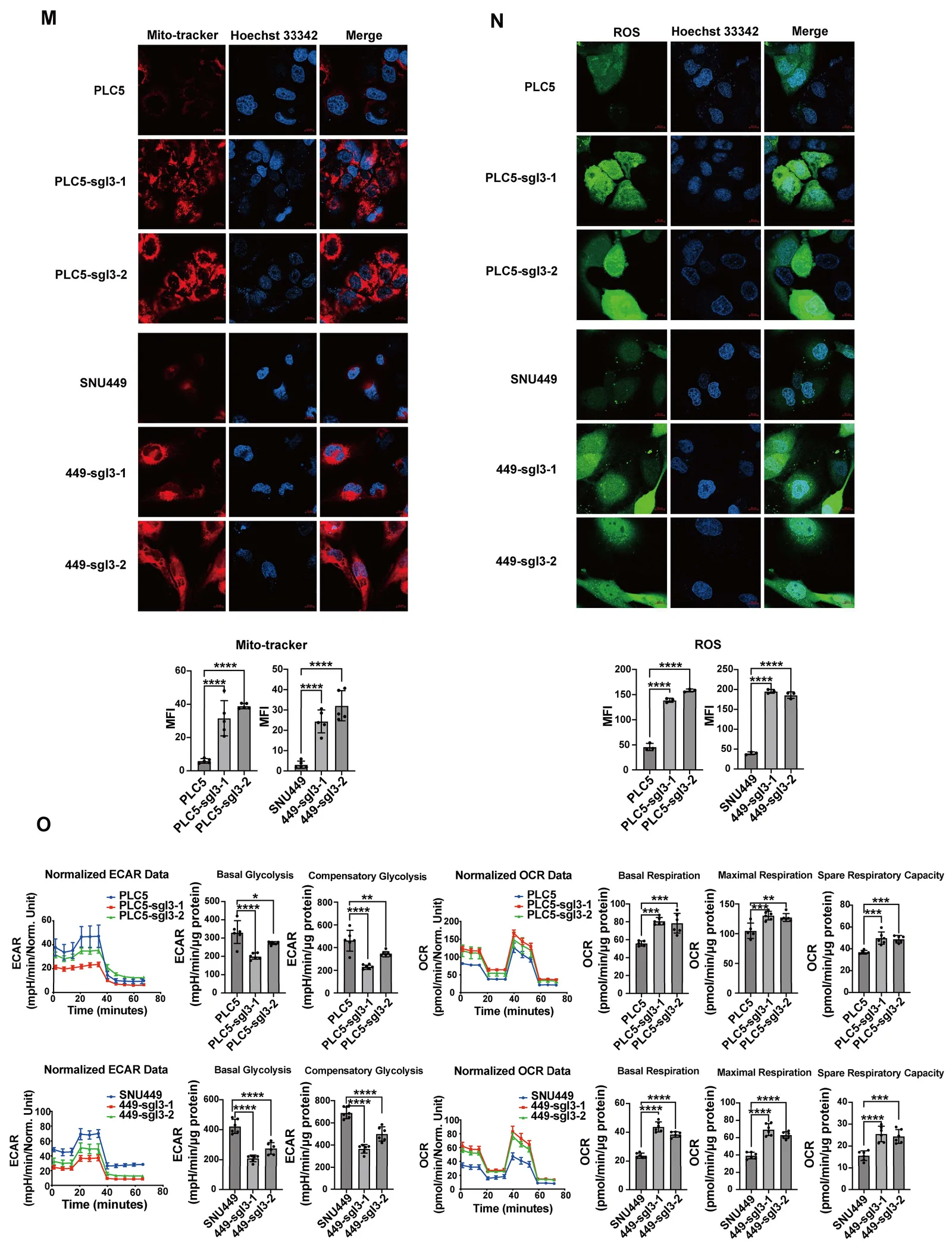
Functional and metabolic characterization of IGF2BP3 in HCC cells. (**A**) GSEA of TCGA-LIHC transcriptomic data stratified by *IGF2BP3* expression levels. The red box highlights the “Hallmark Glycolysis” pathway. (**B**) Overexpression efficiency validated by Western blot and qRT-PCR in SNU387 and PLC/PRF/5 cells. (**C**) CCK-8, colony formation, and Transwell migration assays in *IGF2BP3* overexpressing versus control cells. (**D**) Quantification of glycolytic intermediates (G6P, lactate) and redox ratios (NADP^+^/NADPH, GSSG/GSH) in overexpressing cells. (**E**) Representative fluorescence images and quantitative analysis of mitochondrial mass/charge (Mito-tracker) in overexpressing cells. (**F**) Quantification of intracellular ROS levels in overexpressing cells. (**G**) qPCR of mtDNA encoded genes (*TL1*, *COX1*) normalized to *VDAC1*. (**H**) Seahorse extracellular flux analysis of ECAR and OCR for glycolytic and mitochondrial respiration. (**I**) Knockout efficiency assessed by Western blot and qRT-PCR in CRISPR-Cas9-edited cells. (**J**) Proliferation and migration assays in *IGF2BP3* knockout cell lines. (**K**–**N**) Metabolic and mitochondrial phenotyping in knockout cells, including quantification of G6P/lactate, redox ratios, qPCR of mtDNA encoded genes (*TL1*, *COX1*), Mito-tracker staining, and ROS detection. (**O**) Seahorse comparison of ECAR and OCR parameters between knockout and control groups. “Norm. Unit” indicates the “/μg protein” (per μg protein), as in the bar graphs (* *p* < 0.05, ** *p* < 0.01, *** *p* < 0.001, **** *p* < 0.0001). Scale bar: 10 μm for (**E**,**F**,**M**,**N**); 100 μm for (**C**,**J**).

To elucidate the role of IGF2BP3 in HCC, we first stratified TCGA-LIHC patients into high- and low-expression groups by median *IGF2BP3* expression. Differentially expressed genes between groups revealed significant enrichment of glycolysis signaling by gene set enrichment analysis (GSEA) (Figure 2A). Functional assays confirmed enhanced proliferation, colony formation, and migration in PLC/PRF/5 and SNU387 cells stably overexpressing *IGF2BP3* (Figure 2B,C). To investigate metabolic reprogramming, we measured key glycolytic and redox parameters in these cells. Overexpression of *IGF2BP3* led to elevated glucose-6-phosphate (G6P) and lactate levels, coupled with decreased NADP^+^/NADPH and GSSG/GSH ratios, indicating an enhanced glycolytic flux coupled with increased pentose phosphate pathway (PPP) activity, resulting in the accumulation of reducing equivalents (primarily NADPH and GSH) (Figure 2D). Mito-tracker staining revealed reduced mitochondrial mass/charge (Figure 2E), corroborated by decreased mitochondrial gene copy numbers (*TL1* and *COX1*, Figure 2G). Intracellular ROS levels were also attenuated (Figure 2F), while Seahorse analysis showed increased extracellular acidification rate (ECAR) and decreased oxygen consumption rate (OCR), consistent with a glycolytic dependency (Figure 2H).

Conversely, CRISPR-Cas9-mediated knockout of *IGF2BP3* in PLC/PRF/5 and SNU449 cells (Figure 2I and Figure S2) significantly suppressed proliferation and migration (Figure 2J). Knockout of *IGF2BP3* reduced G6P and lactate, elevated NADP^+^/NADPH and GSSG/GSH ratios, restored mitochondrial gene copies and Mito-tracker fluorescence, and increased ROS production (Figure 2K–N). Seahorse analysis further confirmed decreased ECAR and increased OCR upon knockout (Figure 2O). Collectively, these findings establish that IGF2BP3 drives metabolic reprogramming in HCC by impairing mitochondrial function and enhancing glycolysis.

### 2.3. IGF2BP3 Mediates Metabolic Reprogramming in HCC Through PLK1 Regulation

#### 2.3.1. Co-Upregulation of IGF2BP3 and Its Target PLK1 Predicts Poor Prognosis in HCC

**Figure 3:**
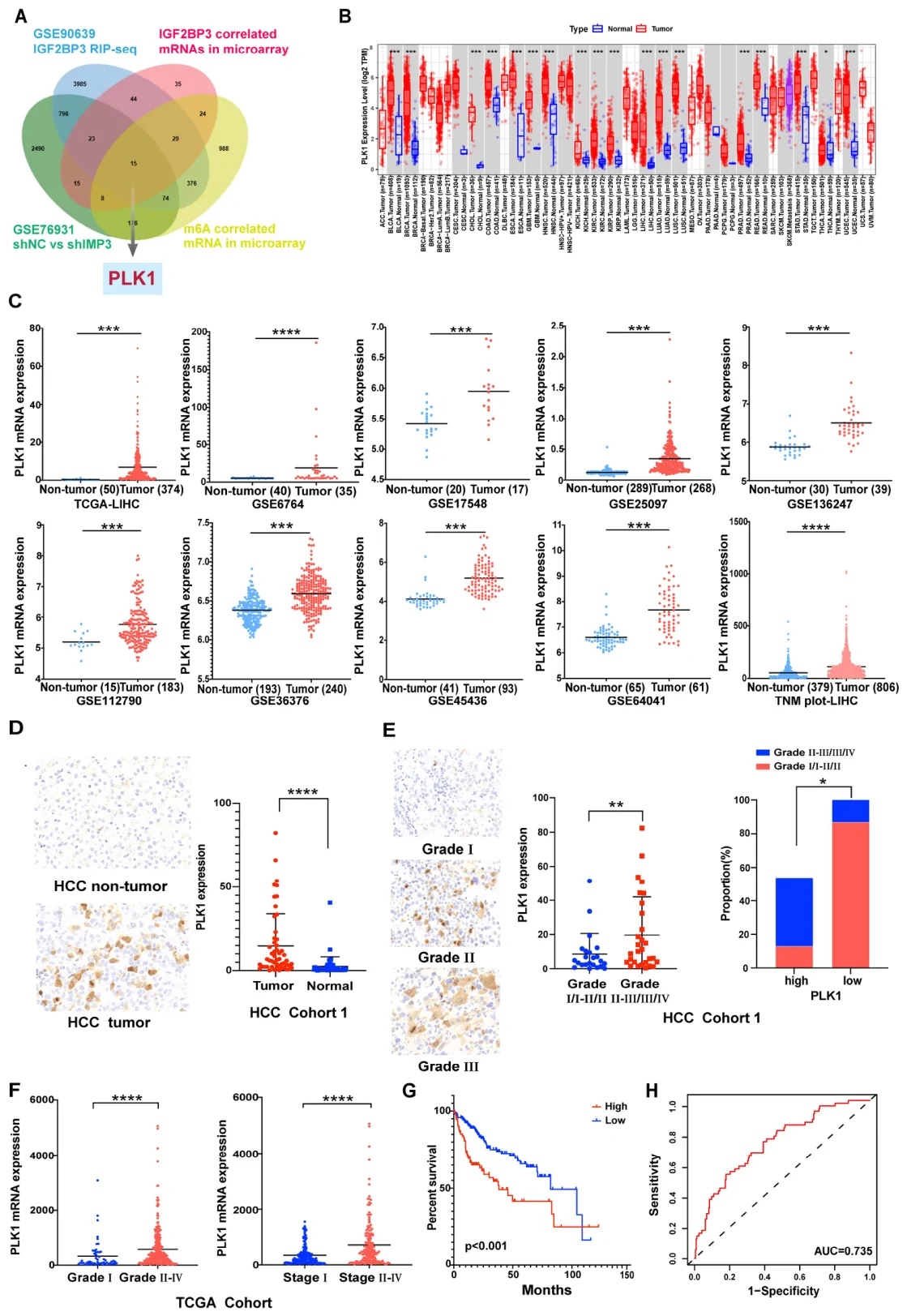

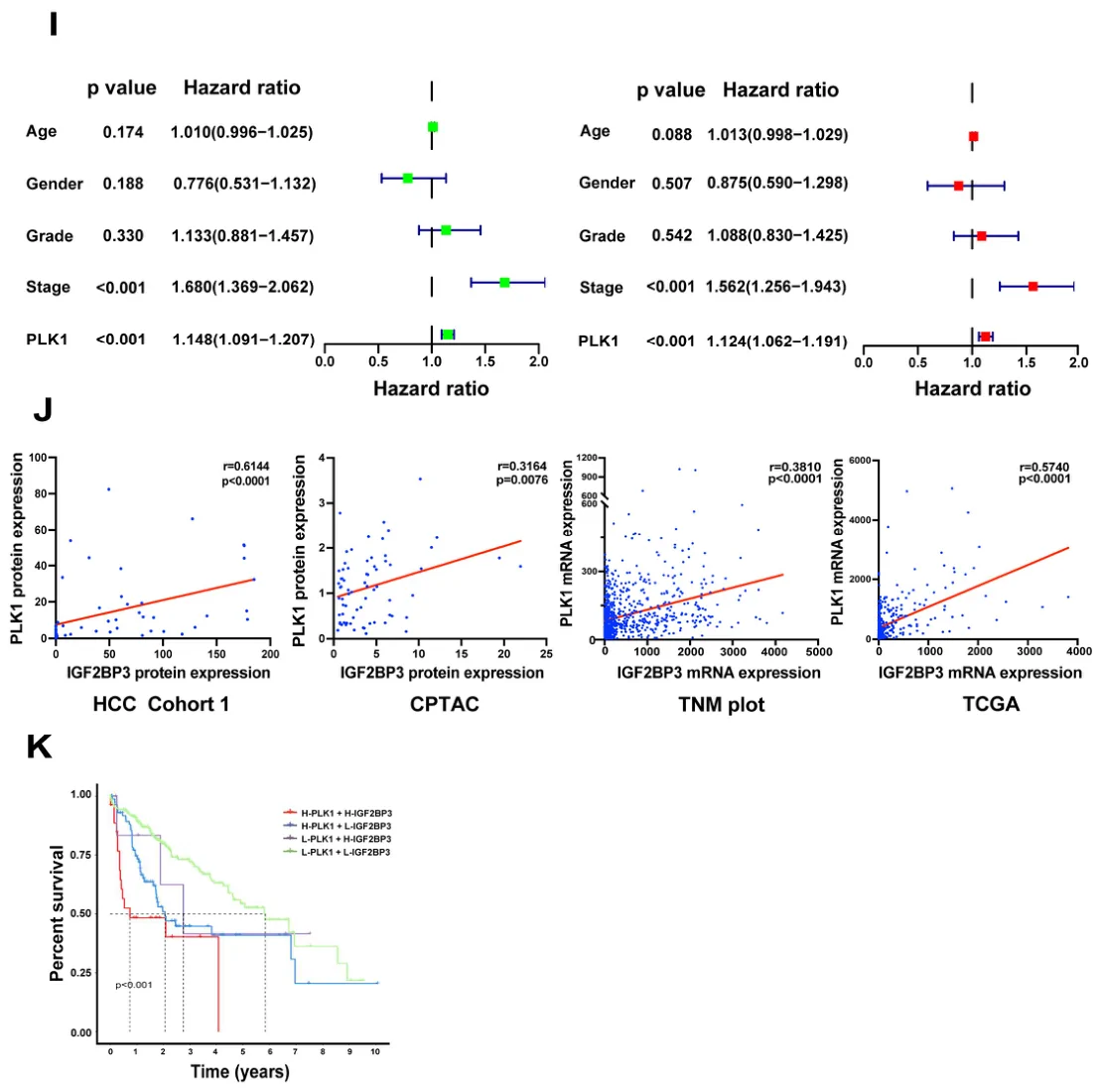
Multi-omics characterization of the IGF2BP3-PLK1 regulatory axis and its clinical relevance in HCC. (**A**) Venn diagram illustrating the intersection of candidate genes from GSE90639 (IGF2BP3 RIP-seq), GSE76931 (*IGF2BP3* knockdown DEGs), m6A-associated mRNA in microarray, and *IGF2BP3*-associated mRNA in microarray. (**B**) Pan-cancer analysis of *PLK1* mRNA across various solid tumor using (TCGA). Red indicates tumor samples and blue indicates normal samples. (**C**) *PLK1* mRNA levels in HCC versus adjacent normal tissues across multiple cohorts (TCGA-LIHC, GEO, TNMplot). (**D**) IHC staining of PLK1 in HCC tumor tissues and adjacent non-tumor tissues (HCC cohort 1). (**E**) *PLK1* expression by tumor grade (I–III) and grade wise distribution in Cohort 1. (**F**) *PLK1* mRNA expression by grade and stage in TCGA-LIHC. (**G**) Kaplan–Meier curves for OS stratified by *PLK1* expression. (**H**) ROC curve for survival prediction using *PLK1*. The diagonal dashed line indicates the random reference line. (**I**) Univariate and multivariate Cox proportional hazards regression analyses evaluating the prognostic association of *PLK1* and clinical factors with overall survival. The vertical dashed line marks the null reference at hazard ratio = 1. (**J**) Pearson correlation between *IGF2BP3* and *PLK1* expression across multiple datasets (Cohort 1, CPTAC, TNM plot, and TCGA). (**K**) Kaplan–Meier curves for OS stratified by combined *IGF2BP3/PLK1* expression in HCC patients. The horizontal dashed line denotes the 50% survival reference for median survival estimation (* *p* < 0.05, ** *p* < 0.01, *** *p* < 0.001, **** *p* < 0.0001).

To identify physiologically relevant downstream targets of the m6A reader IGF2BP3, we performed an integrative analysis of multiple public datasets. Specifically, we intersected genes identified by IGF2BP3 RIP-seq (GSE90639) with those differentially expressed following *IGF2BP3* knockdown (GSE76931), and further overlapped this common set with both m6A-modified and *IGF2BP3*-associated transcripts from microarray profiling. This sequential filtering reduced the initial gene pools to a shortlist of 15 candidates. Among these, *PLK1* emerged as the leading candidate, as prioritized by literature evidence (Figure 3A).

Pan-cancer analysis of TCGA data revealed significant *PLK1* overexpression across multiple solid tumors relative to adjacent normal tissues (Figure 3B). This was further validated in HCC by integrated TCGA/GEO/TNMplot cohorts at mRNA/protein levels (Figure 3C) and by IHC on tissue microarrays (Figure 3D). Clinically, *PLK1* positively associated with tumor grade: high-grade tumors displayed higher levels, and high-PLK1 patients more often had advanced pathological grades (Figure 3E). TCGA transcriptomic data also showed progressive mRNA increases with grade and stage (Figure 3F). High *PLK1* expression correlated with shorter overall survival (Figure 3G), and ROC analysis indicated prognostic performance for survival prediction (Figure 3H). Multivariate Cox regression adjusted for established clinical covariates demonstrated an independent prognostic association of *PLK1* with overall survival in the TCGA-LIHC cohort (Figure 3I). To investigate upstream regulation, multi-omics integration from TCGA, TNMplot, CPTAC, and tissue microarrays revealed a strong positive association between *IGF2BP3* and *PLK1* at both mRNA and protein levels (Figure 3J). Combined expression analysis demonstrated that double-high patients had the shortest OS, double-low the best, and single-high intermediate survival (Figure 3K). In single-cell transcriptomic data, *IGF2BP3* and *PLK1* were preferentially detected in malignant cells, and their elevated levels—particularly in the double-high group—tracked with a larger malignant compartment, implying functional synergy (Figure S1).

#### 2.3.2. IGF2BP3 Enhances PLK1 Stability Through m6A Modification

**Figure 4:**
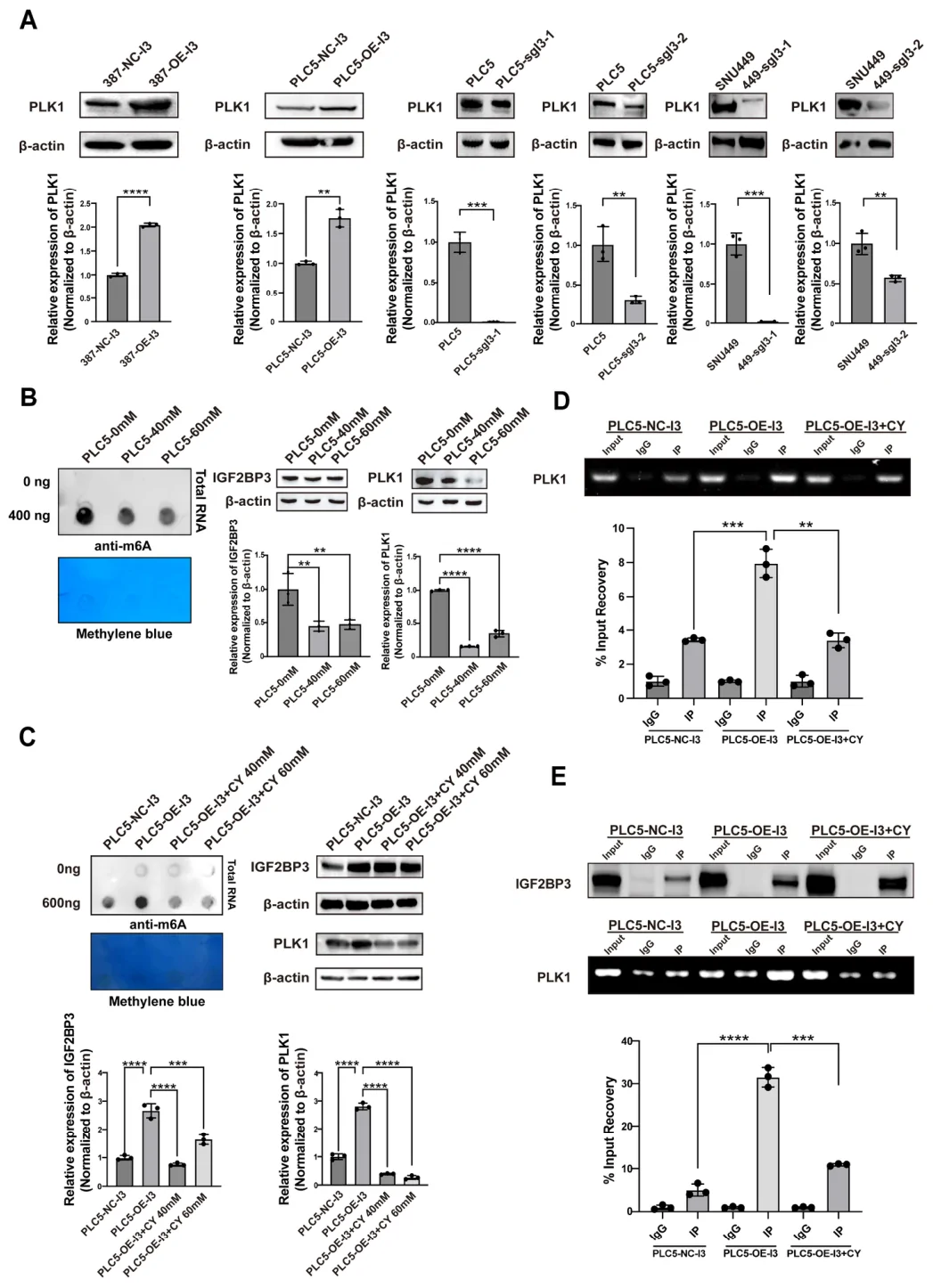

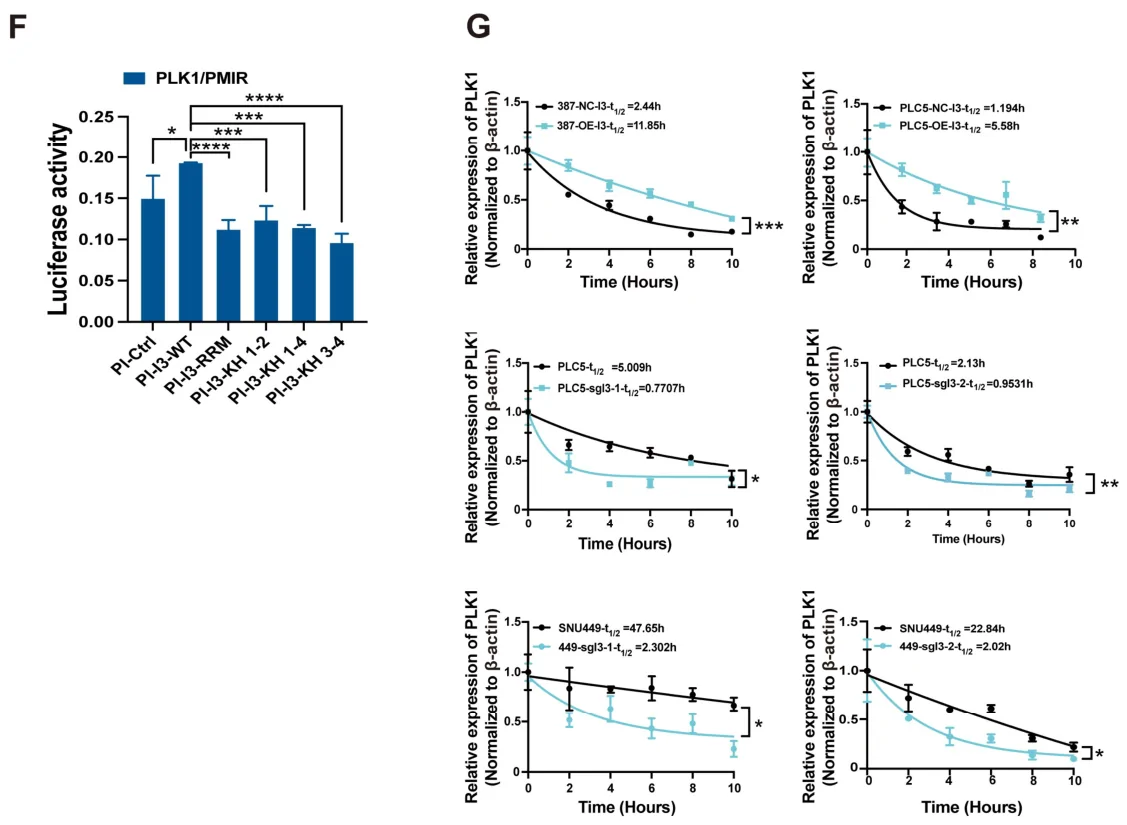
IGF2BP3 regulates *PLK1* expression via m6A modification. (**A**) PLK1 mRNA and protein levels following *IGF2BP3* overexpression or knockout in PLC/PRF/5 and SNU449 cells. (**B**) Dot blot for global m6A, Western blot for IGF2BP3 and PLK1, and qRT-PCR for *PLK1* mRNA upon treatment with cycloleucine. (**C**) *PLK1* expression (qRT-PCR and Western blot) in *IGF2BP3* overexpressing cells treated with cycloleucine. (**D**) MeRIP qRT-PCR analysis of m6A enrichment on *PLK1* mRNA under *IGF2BP3* overexpression and cycloeucine treatment. (**E**) RIP-qRT-PCR assessing direct binding of IGF2BP3 to *PLK1* mRNA. (**F**) Luciferase reporter assay measuring *PLK1* luciferase activity in cells transfected with wild type or mutant IGF2BP3 (lacking RRM and KH domains). (**G**) Actinomycin D chase assay for *PLK1* mRNA stability and half-life in cells with *IGF2BP3* overexpression or knockout. “CY” means Cycloleucine, it was used at final concentrations of 40 mM and 60 mM for 24 h, with ddH_2_O-treated cells serving as controls. Data are presented as mean ± SD from at least three independent experiments (* *p* < 0.05, ** *p* < 0.01, *** *p* < 0.001, **** *p* < 0.0001).

To elucidate the regulatory mechanism of the IGF2BP3-PLK1 axis, we modulated *IGF2BP3* expression in HCC cells. Overexpression upregulated *PLK1* at both mRNA and protein levels, whereas knockdown reduced its expression (Figure 4A), confirming a positive regulatory role. Treatment with cycloleucine (S-adenosylmethionine-mediated methylation inhibitor) decreased global m6A levels (dot-blot) without affecting IGF2BP3 protein level, yet markedly reduced *PLK1* expression (Figure 4B); importantly, cycloleucine also abrogated the *IGF2BP3*-induced upregulation of *PLK1* (Figure 4C). MeRIP-qRT-PCR revealed specific m6A enrichment of *PLK1* transcripts, which was enhanced by *IGF2BP3* overexpression and attenuated by cycloeucine treatment (Figure 4D), implicating that *IGF2BP3* overexpression was associated with increased m6A enrichment of *PLK1* mRNA. Meanwhile RIP-qRT-PCR confirmed direct binding of IGF2BP3 to *PLK1* mRNA, with increased affinity following *IGF2BP3* overexpression (Figure 4E). Luciferase reporter assays showed that wild-type IGF2BP3 significantly enhanced *PLK1* reporter activity, whereas mutational dysfunction of RRM and KH domains abolished this effect (Figure 4F). Actinomycin D chase assays demonstrated that *IGF2BP3* overexpression prolonged the half-life of *PLK1* mRNA, whereas *IGF2BP3* depletion had the opposite effect (Figure 4G). Collectively, these findings indicate that IGF2BP3 may enhance the stability of m6A-modified *PLK1* transcripts, thereby increasing *PLK1* expression level.

#### 2.3.3. PLK1 Drives Glycolysis While Suppressing Oxidative Phosphorylation in HCC

**Figure 5:**
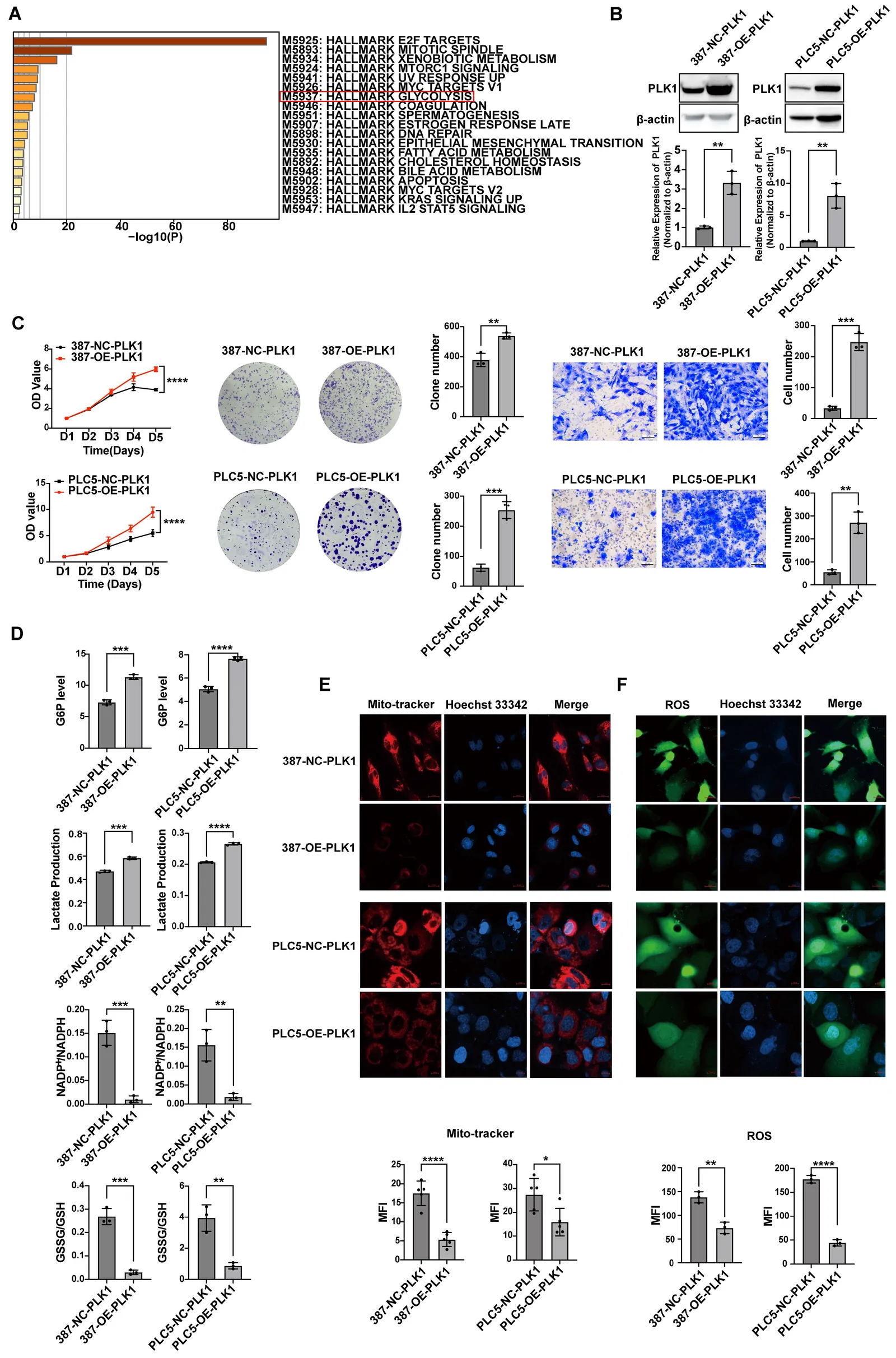

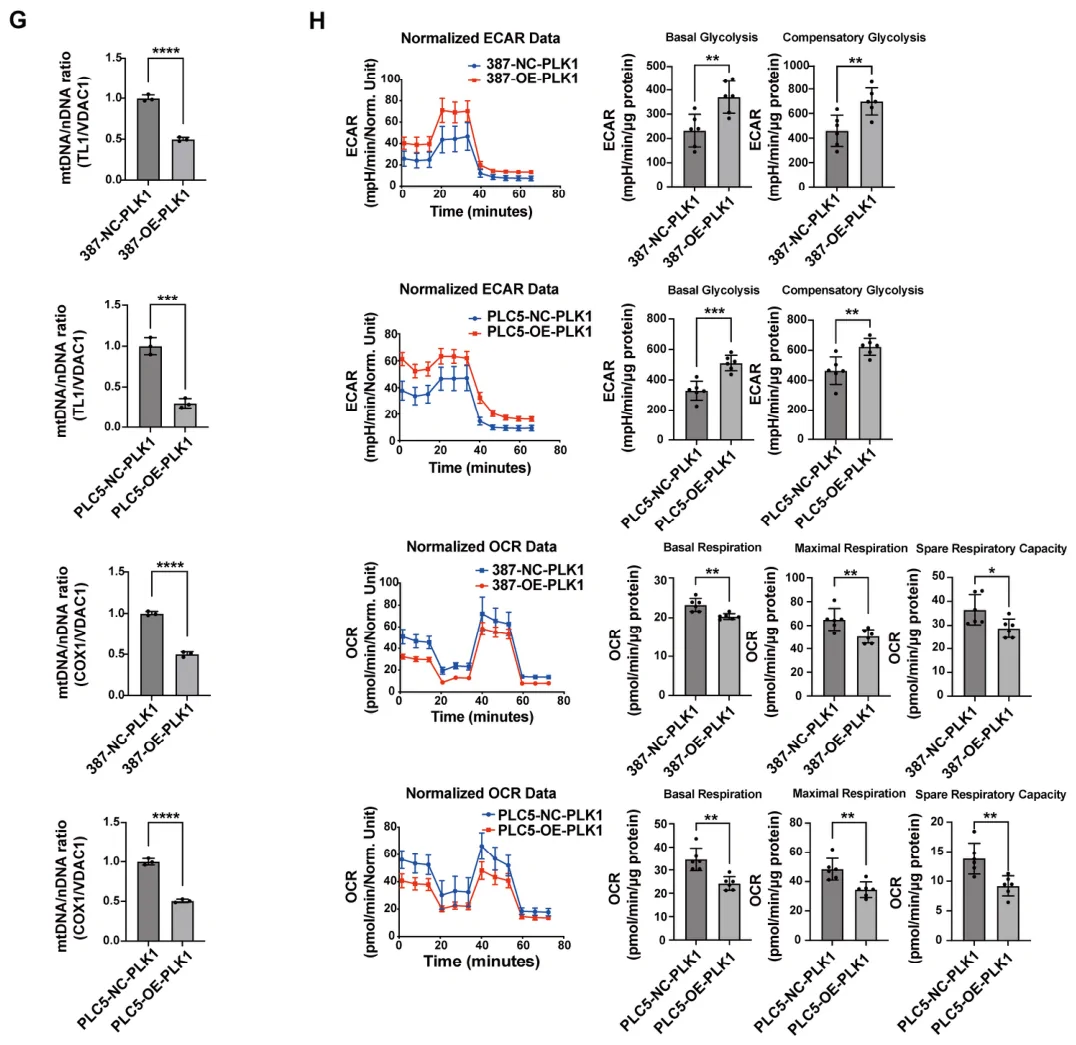

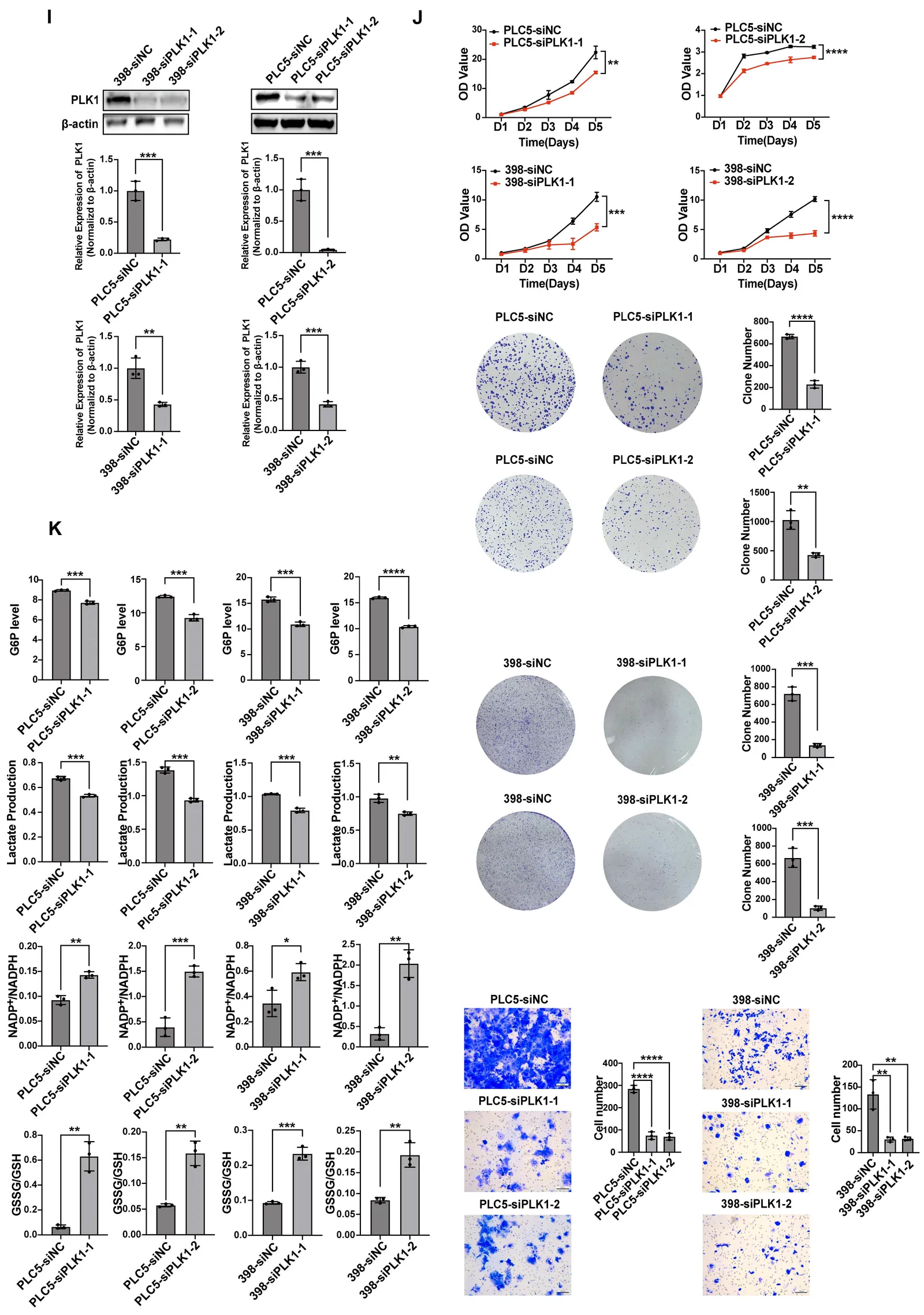

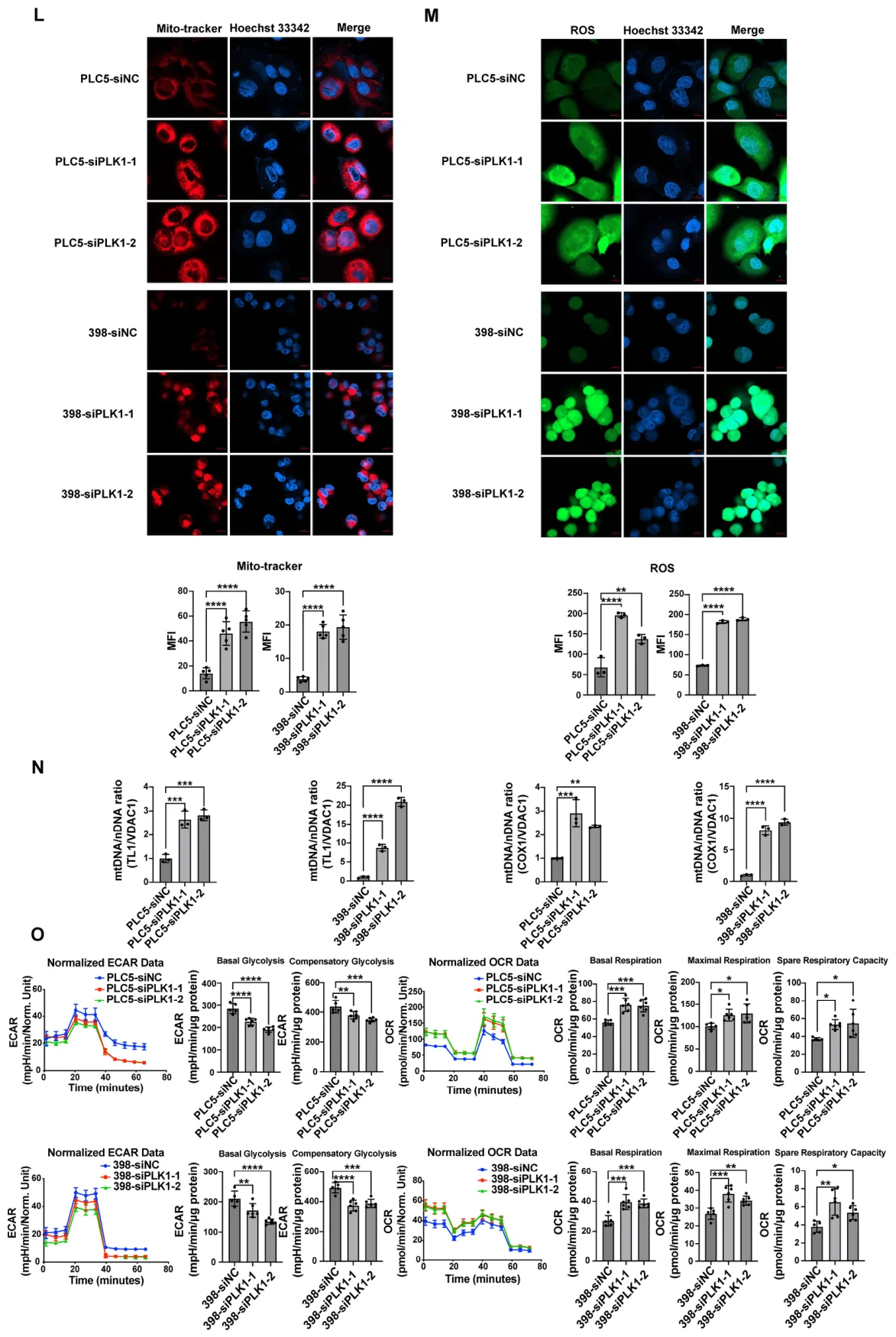
*PLK1*-mediated biological functions and metabolic alterations in HCC. (**A**) Hallmark pathway enrichment of DEGs stratified by median *PLK1* expression in TCGA HCC patients. The red box highlights the “Hallmark Glycolysis” pathway. (**B**) *PLK1* overexpression verified by Western blot and qRT-PCR in PLC/PRF/5 and SNU387 cells. (**C**) Cell proliferation and migration assays in *PLK1*-overexpressing cells. (**D**–**H**) Metabolic and mitochondrial profiling in *PLK1*-overexpressing cells: G6P/lactate levels, redox ratios (NADP^+^/NADPH, GSSG/GSH), Mito-tracker staining for mitochondrial mass/charge, ROS levels, qPCR of mtDNA encoded genes (*TL1* and *COX1*) and Seahorse ECAR/OCR. “Norm. Unit” indicates the “/μg protein” (per μg protein), as in the bar graphs. (**I**) *PLK1* knockdown verified by Western blot and qRT-PCR in PLC/PRF/5 and SNU398 cells. (**J**) Proliferation and migration assays in knockdown cells. (**K**–**O**) Corresponding metabolic and mitochondrial analyses in knockdown cells (same parameters as (**D**–**H**)) (* *p* < 0.05, ** *p* < 0.01, *** *p* < 0.001, **** *p* < 0.0001). Scale bar: 10 μm for (**E**,**F**,**L**,**M**); 100 μm for (**C**,**J**).

To investigate the role of *PLK1* in HCC progression, TCGA patients were stratified by median *PLK1* expression. Differential expression and hallmark pathway enrichment analyses revealed significant association of *PLK1* with glycolysis (Figure 5A). We therefore established stable *PLK1*-overexpressing PLC/PRF/5 and SNU387 cells (Figure 5B), which exhibited markedly enhanced proliferation and migration in CCK-8, colony formation, and Transwell assays (Figure 5C).

Metabolic profiling of *PLK1*-overexpressing cells showed increased G6P and lactate levels, reduced NADP^+^/NADPH and GSSG/GSH ratios (Figure 5D), diminished Mito-tracker fluorescence and ROS intensity (Figure 5E,F), and decreased *TL1* and *COX1* copy numbers (Figure 5G). Seahorse analysis further indicated elevated ECAR and reduced OCR (Figure 5H). Conversely, *PLK1* knockdown in PLC/PRF/5 and SNU398 cells (Figure 5I) suppressed proliferation and migration (Figure 5J). At the metabolic level, knockdown resulted in decreased G6P and lactate, increased NADP^+^/NADPH and GSSG/GSH ratios, elevated *TL1* and *COX1* copies, and enhanced Mito-tracker and ROS signals (Figure 5K–N), along with reduced ECAR and increased OCR (Figure 5O). Collectively, these findings demonstrate that *PLK1* drives a metabolic rewiring from oxidative phosphorylation to glycolysis, thereby promoting HCC progression.

### 2.4. The IGF2BP3-m6A-PLK1 Axis Orchestrates Metabolic Reprogramming in HCC

**Figure 6:**
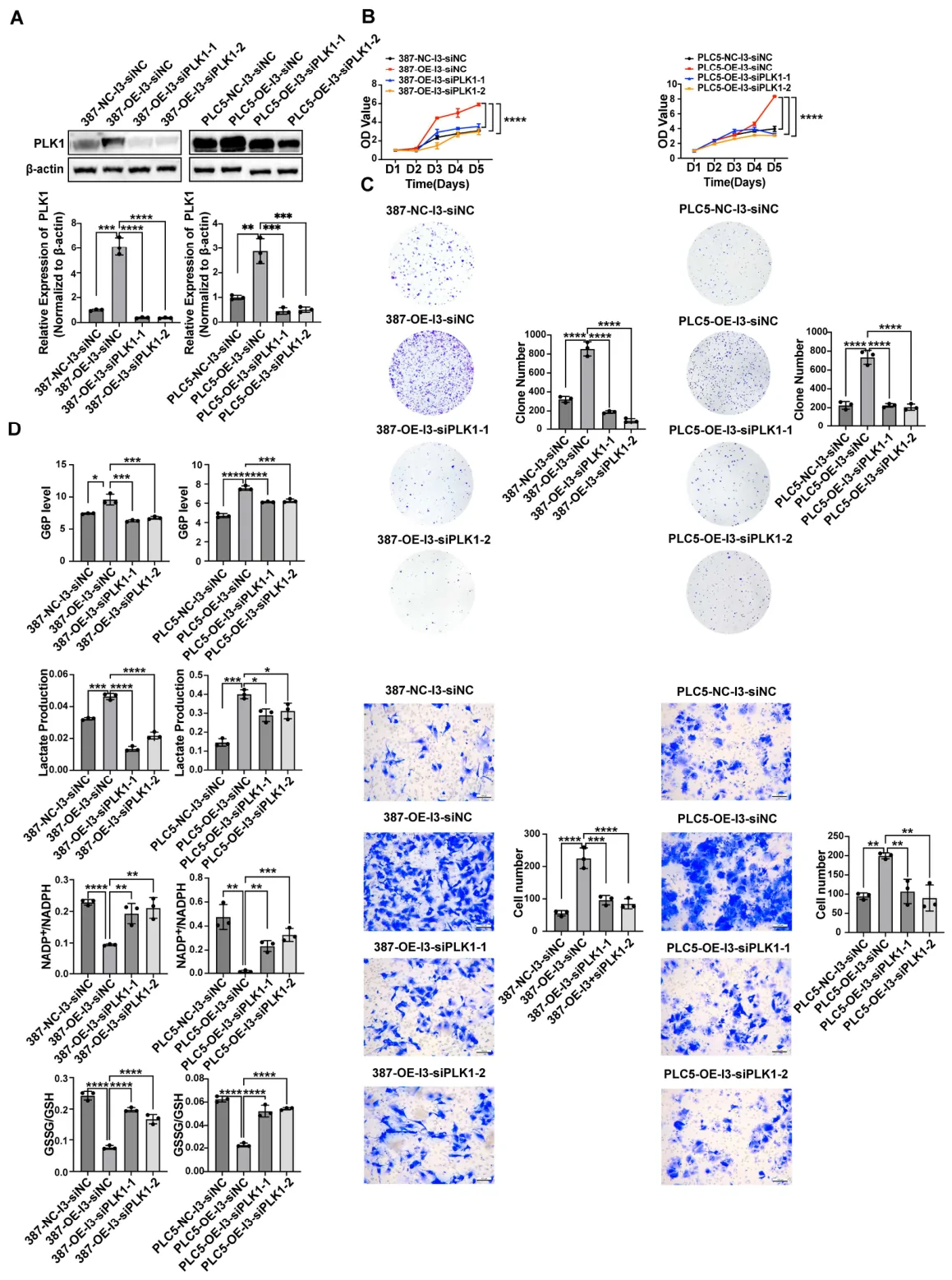

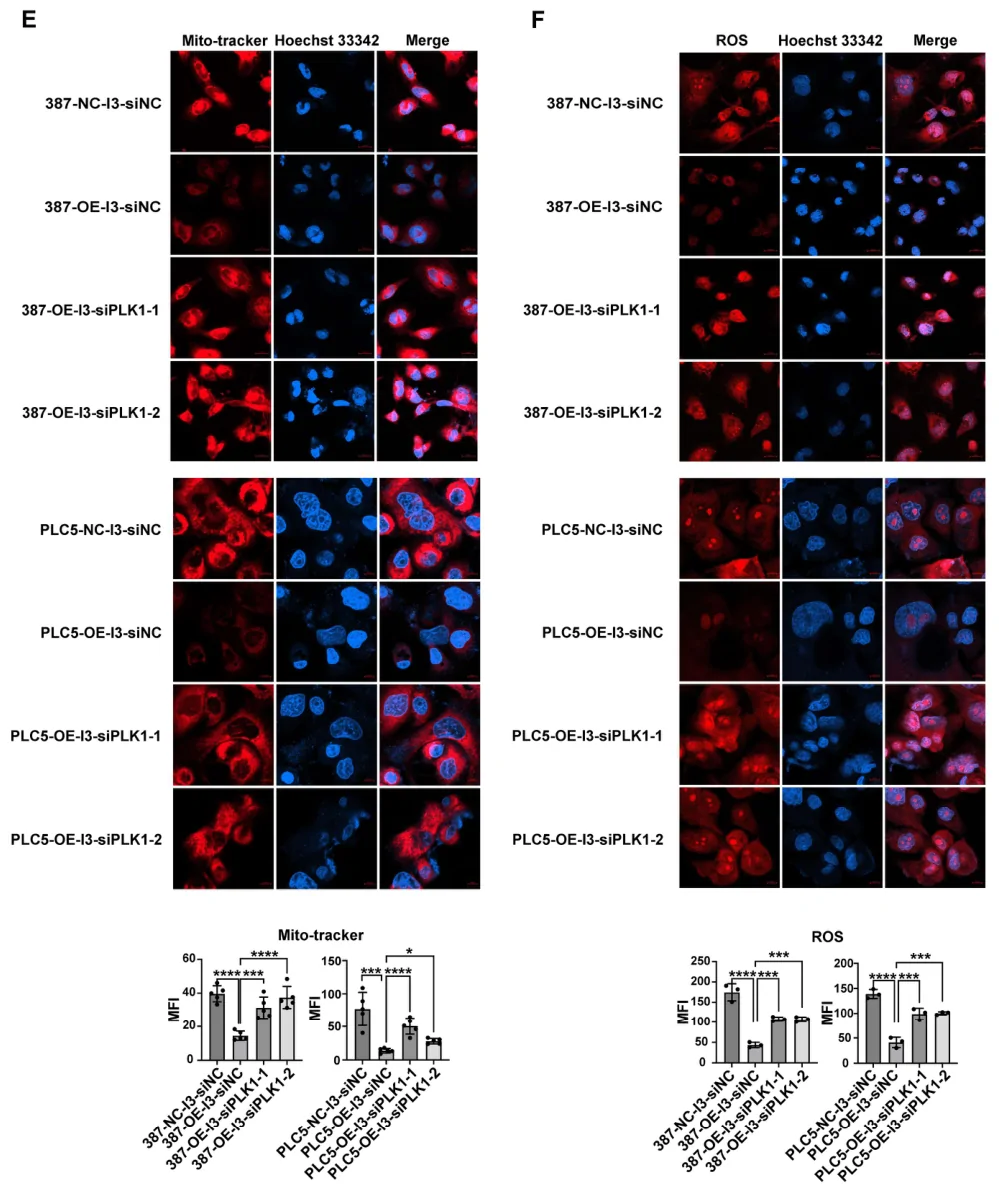

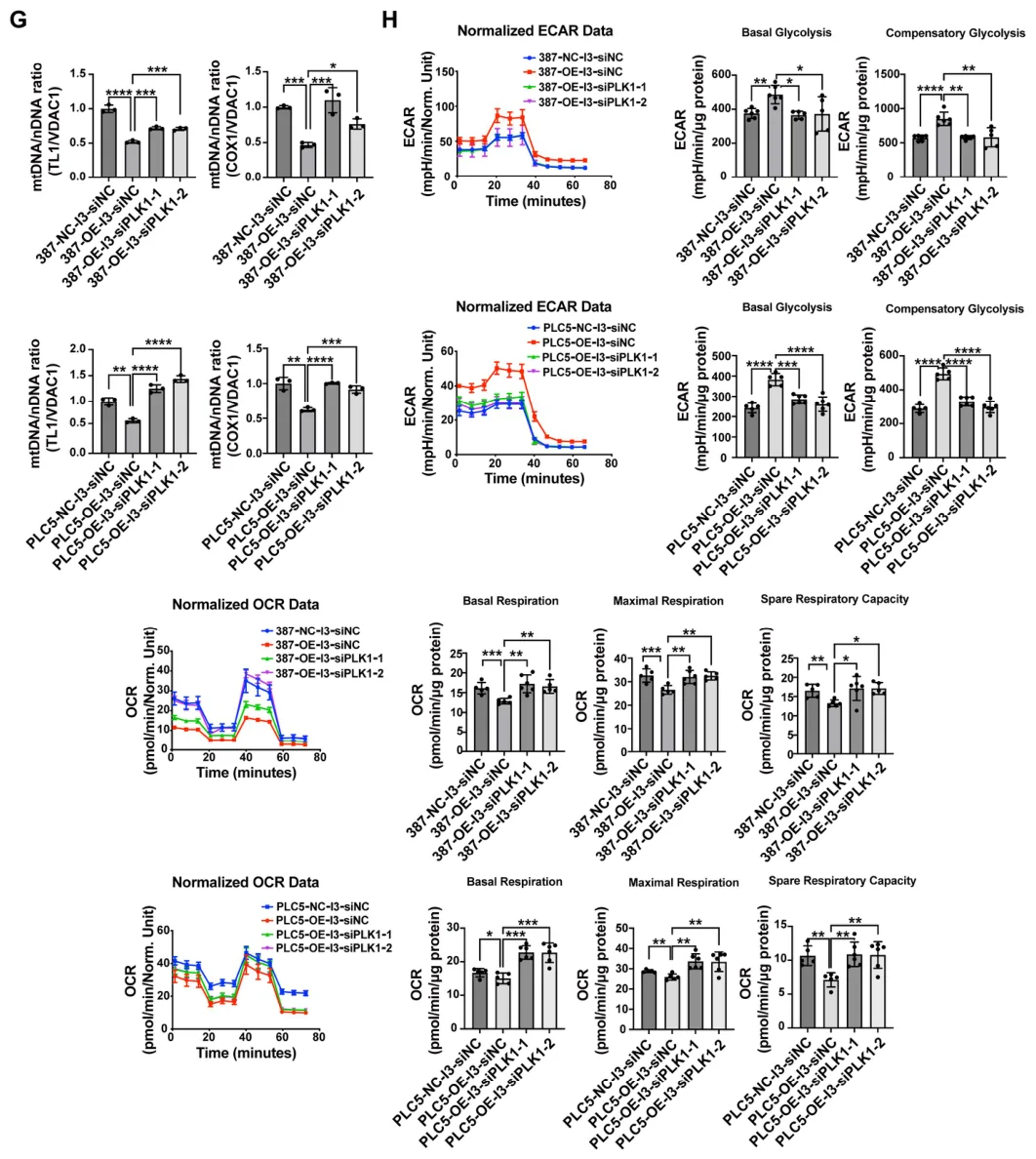

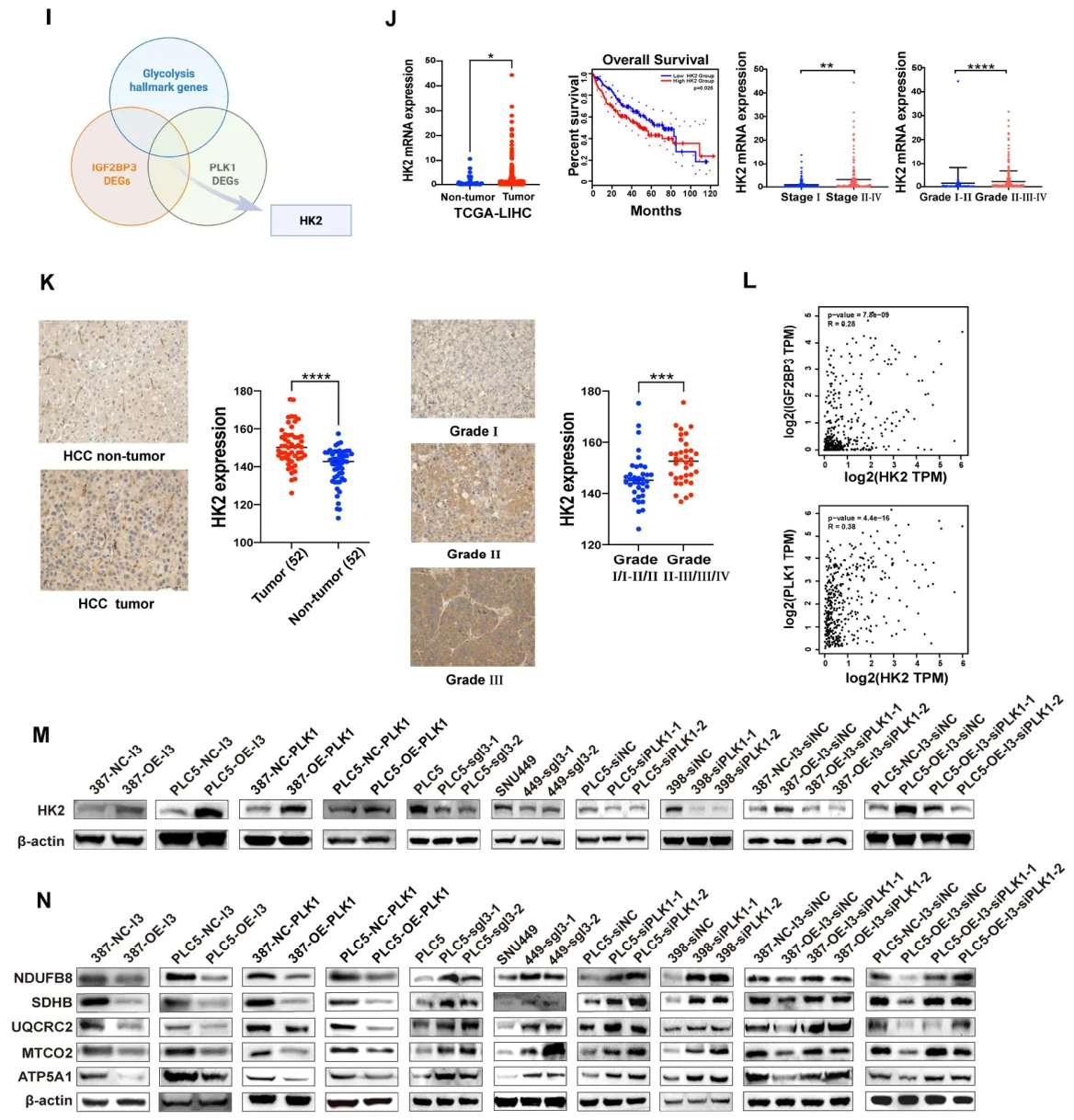
Validation of the IGF2BP3-PLK1-HK2 axis in HCC metabolic reprogramming. (**A**) *PLK1* knockdown verified by Western blot and qRT-PCR in *IGF2BP3* overexpressing cells. (**B**,**C**) Cell proliferation and migration assays in rescue models. (**D**–**H**) Metabolic and mitochondrial phenotyping in rescue models, including G6P/lactate, redox ratios, qPCR of mtDNA encoded genes (*TL1* and *COX1*), Mito-tracker, ROS, and ECAR/OCR. “Norm. Unit” indicates the “/μg protein” (per μg protein), as in the bar graphs. (**I**) Bioinformatic identification of *HK2* as a key glycolytic gene using TCGA data and MSigDB hallmark gene sets. (**J**) Clinical association analysis of *HK2* expression with patient survival, tumor stage, and grade. (**K**) IHC staining of HK2 in HCC tissue microarrays. (**L**) Association of *HK2* with *IGF2BP3* and *PLK1* expression in TCGA. (**M**) Western blot for HK2 protein expression in *IGF2BP3/PLK1*-modulated cells. (**N**) Western blot for mitochondrial electron transport chain complex subunits (NDUFB8, SDHB, UQCRC2, MTCO2, ATP5A1) in *IGF2BP3/PLK1*-modulated cells (* *p* < 0.05, ** *p* < 0.01, *** *p* < 0.001, **** *p* < 0.0001). Scale bar: 10 μm for (**E**,**F**); 100 μm for (**C**).

To validate *PLK1* as a critical downstream effector of IGF2BP3-driven metabolic reprogramming, we performed rescue experiments in SNU387 and PLC/PRF/5 cells stably overexpressing *IGF2BP3*, with subsequent *PLK1* knockdown via siRNA (Figure 6A). Functional assays showed that *PLK1* depletion significantly reversed the IGF2BP3-induced enhancement of proliferation and migration (Figure 6B,C). Metabolically, *PLK1* knockdown rescued the IGF2BP3-mediated Warburg effect, as evidenced by decreased G6P and lactate, restored NADP^+^/NADPH and GSSG/GSH ratios, increased Mito-tracker and ROS fluorescence, and elevated *TL1* and *COX1* copy numbers (Figure 6D–G). Seahorse analysis further confirmed that *PLK1* silencing reversed the IGF2BP3-induced increase in ECAR and decrease in OCR, shifting metabolism back toward oxidative phosphorylation (Figure 6H). Collectively, these results establish *PLK1* as a key downstream mediator through which *IGF2BP3* drives metabolic reprogramming and malignancy in HCC.

To decipher the molecular basis, we intersected glycolysis hallmark genes (MSigDB) with differentially expressed genes stratified by median *IGF2BP3* and *PLK1* expression in TCGA, identifying *HK2* as a pivotal glycolytic gene (Figure 6I). Clinical association analyses revealed *HK2* upregulation in HCC tissues, with increasing mRNA levels across advanced clinical stages and pathological grades, and a strong negative association with prognosis (Figure 6J). Tissue microarray IHC confirmed elevated HK2 protein in 52 HCC tumors, positively associated with pathological grade (Figure 6K), and *HK2* transcription significantly associated with both *IGF2BP3* and *PLK1* expression (Figure 6L). Western blotting showed that *IGF2BP3/PLK1* overexpression upregulated HK2 protein, while their knockout or knockdown suppressed it (Figure 6M). Concurrently, examination of mitochondrial electron transport chain complexes I–V (NDUFB8, SDHB, UQCRC2, MTCO2, ATP5A1) revealed that *IGF2BP3/PLK1* overexpression downregulated these complexes, whereas their depletion restored complex levels (Figure 6N). Thus, the IGF2BP3-PLK1 axis may suppress mitochondrial respiratory chain complexes, impairing OXPHOS and driving metabolic reprogramming by upregulating HK2.

**Figure 7:**
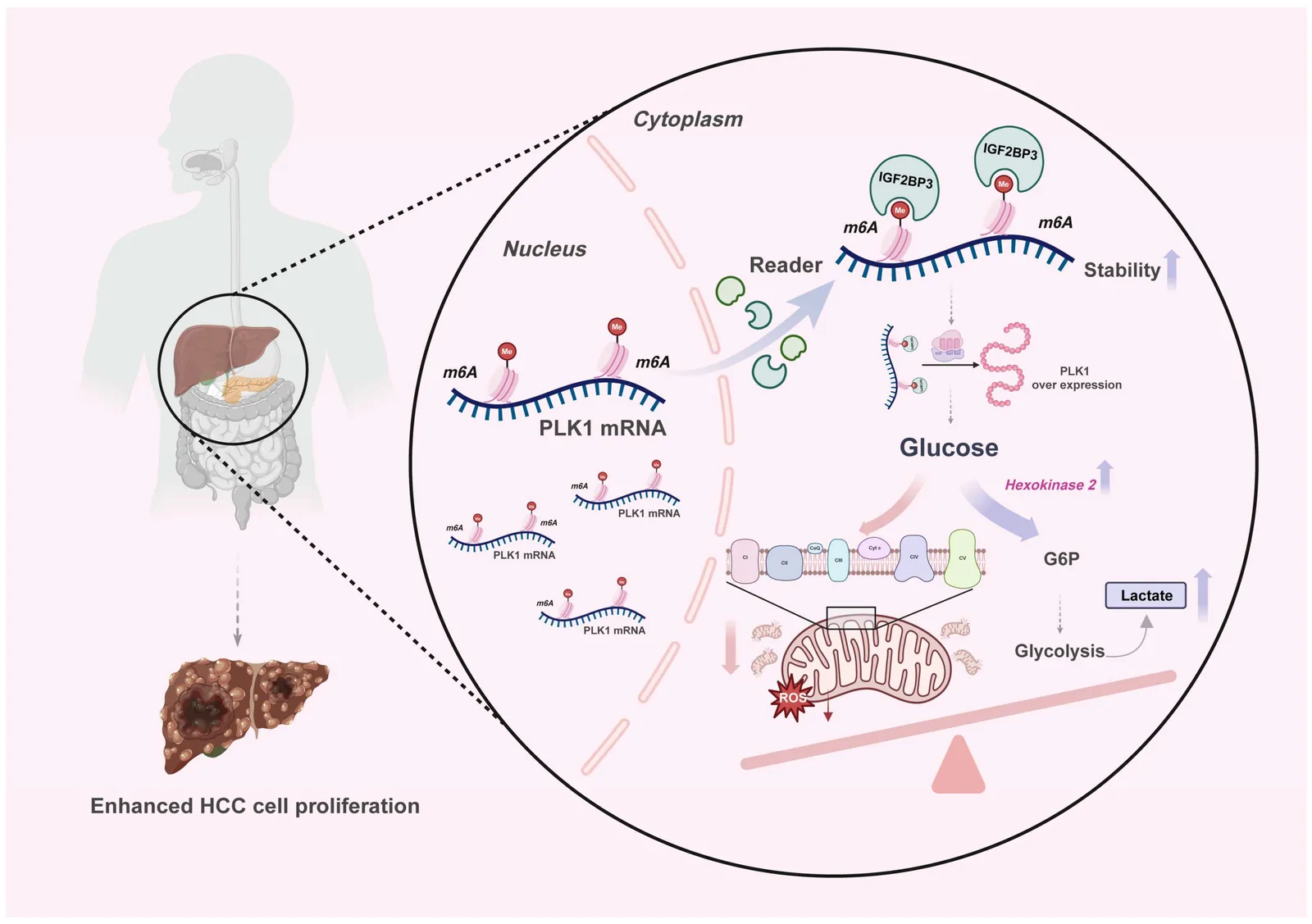
Schematic illustration of the mechanism by which IGF2BP3 promotes HCC progression via stabilizing m6A-modified *PLK1* mRNA and driving metabolic reprogramming.

In summary, IGF2BP3 may recognize and bind m6A-modified *PLK1* transcripts to enhance *PLK1* stability. This axis suppresses mitochondrial electron transport and concomitantly drives the Warburg effect, thereby accelerating HCC malignant progression (Figure 7).

## 3. Discussion

In this study, we identified the IGF2BP3-PLK1 axis as a critical driver of malignant progression and metabolic reprogramming in HCC. Herein, we demonstrated that IGF2BP3 stabilizes m6A-modified *PLK1* mRNA, thereby promoting aerobic glycolysis and suppressing mitochondrial OXPHOS. This metabolic rewiring is likely mediated through the upregulation of HK2, the rate-limiting switch enzyme catalyzing the first step of glycolysis [22,23]. These findings significantly expand our understanding of how epitranscriptomic regulation dictates cancer metabolism and highlight the oncogenic synergy between IGF2BP3 and *PLK1* in HCC.

In HCC, *PLK1* has been identified as an essential regulator of aerobic glycolysis [20]. Although *PLK1*-dependent glycolysis is not a novel finding per se, the identification of IGF2BP3 as an upstream regulator that governs the expression level of m6A-modified *PLK1* represents a significant conceptual advance. Our study established a functional link between IGF2BP3-mediated regulation and m6A-modified *PLK1*-driven metabolic reprogramming in HCC, thereby connecting two previously separate fields—m6A epitranscriptomics and *PLK1*-mediated metabolism—into a unified regulatory axis.

*IGF2BP3* overexpression increased m6A enrichment on *PLK1* in the MeRIP data of this study. IGF2BP3 was classically regarded as an m6A reader that enhances RNA stability, and it is not generally thought to directly deposit m6A. However, as a non-canonical reader, IGF2BP3 may also indirectly promote and alter the level and pattern of m6A modifications on mRNAs, as recently reported in leukemia cells [24]. This may provide a possible explanation for the increased m6A enrichment of *PLK1* observed in *IGF2BP3*-overexpression HCC cells in our study. Since no direct evidence for IGF2BP3-mediated m6A deposition was obtained in this study, we cannot exclude an indirect mechanism. For example, IGF2BP3 may act through other molecules or pathways, such as stabilizing components of the m6A writer complex, suppressing m6A erasers, to enhance *PLK1* m6A modification. Hence, the mechanism underlying the increased *PLK1* m6A enrichment upon *IGF2BP3* overexpression in HCC cells needs to be elucidated. Regardless of whether IGF2BP3 directly or indirectly alters m6A modification of *PLK1* mRNA, its oncogenic function is ultimately mediated by its canonical m6A reader activity—binding to and stabilizing m6A-modified *PLK1* mRNA, thereby enabling downstream function. Therefore, this study did not address whether IGF2BP3 affects the m6A modification of *PLK1* mRNA, instead, it focused on the effect of IGF2BP3 on *PLK1* mRNA stability.

The profound metabolic reprogramming induced by the IGF2BP3-PLK1 axis likely confers multiple survival advantages to HCC cells. The suppression of OXPHOS and reliance on glycolysis may reduce ROS production, thereby protecting cancer cells from oxidative stress-induced apoptosis. Concurrently, enhanced glycolysis inevitably leads to lactate accumulation, which not only limits mitochondrial pyruvate uptake but also acidifies the tumor microenvironment (TME) [25]. Furthermore, based on the single-cell analysis presented in Figure S1, elevated expression of *IGF2BP3* and *PLK1* is intricately associated with an expanded malignant cell compartment within the TME. Informed by our current observations and established knowledge, it is tempting to speculate that the resulting lactate-rich, acidic milieu may further impair cytotoxic T lymphocyte function, promote M2 macrophage polarization, and reinforce an immunosuppressive state [26,27]. Subsequent studies in syngeneic mouse systems are critically needed to unravel the causal link between this axis and the reshaping of the antitumor immune milieu in HCC.

A potential significance of this study is its suggestion that the IGF2BP3–m6A–PLK1 axis might represent a therapeutic target worthy of further exploration. Pan-cancer overexpression and HCC-specific prognostic significance underscore IGF2BP3 as a potential therapeutic target [15,18] A small-molecule inhibitor targeting IGF2BP3 (I3IN-002) has recently been reported (bioRxiv, April 2025), which disrupts IGF2BP3–RNA binding [28]. Although still preclinical, I3IN-002 shows promising efficacy in inhibiting leukemia progression in vitro and in mouse models, thereby providing a compelling rationale for further mechanistic interrogation in B-ALL and other malignancies, particularly HCC.

*PLK1* is overexpressed in multiple cancers, driving proliferation, chemoresistance, and poor prognosis [29,30,31,32]. Normal primary cells exhibit weak dependence on PLK1, whereas cancer cells from various origins are “PLK1-addicted,” providing a rationale for tumor-specific PLK1 inhibition [33]. Early small-molecule inhibitors, such as the ATP analogue BI2536 (which also targets BRD4), induced mitotic arrest and apoptosis but showed limited clinical efficacy, leading to discontinuation after phase I/II trials in solid tumors [34,35]. The next-generation inhibitor volasertib (BI6727) demonstrated improved potency and entered phase III for acute myeloid leukemia (AML), receiving FDA Breakthrough Therapy designation; however, its activity in solid tumors remained modest and associated with some toxicity [36,37,38,39,40,41,42,43]. More recently, the highly selective third-generation inhibitor onvansertib (PCM-075) has shown favorable tolerability and robust antitumor activity in preclinical models and early-phase trials [44,45]. It has received FDA Orphan Drug and Fast Track designations and is currently being evaluated in phase II trials for *KRAS*-mutant colorectal cancer and pancreatic ductal adenocarcinoma, representing the only PLK1 inhibitor still in clinical development for solid tumors [46]. Collectively, these clinical advances underscore the therapeutic potential of PLK1-targeted strategies, both as monotherapy and in combination. Based on our findings, we cautiously propose that PLK1 inhibition might represent a putative therapeutic strategy for HCC. However, this interpretation is speculative and awaits experimental confirmation.

The m6A methylation system comprises methyltransferases, demethylases, and reader proteins [47]. Methyltransferases are essential for depositing the m6A mark. Among them, METTL3 and METTL14 form a heterodimer (the MAC complex) that exhibits weak catalytic activity, whereas WTAP, KIAA1429, and ZC3H13 constitute the MACOM complex, which associates with MAC and potentiates its function [48,49,50,51]. To determine whether *PLK1* mRNA undergoes m6A modification in HCC, MeRIP-qRT-PCR was first performed and confirmed the presence of such modification. However, knockdown or overexpression of *METTL3* failed to significantly influence global m6A level, or alter *PLK1* mRNA or protein levels in HCC cells (Figure S6), presumably because modulating *METTL3* expression alone may not be sufficient to recruit MACOM activity. Another possibility is that the methyltransferase activity of METTL3 is cell-context dependent, which indirectly suggests the complexity of m6A regulatory mechanisms. Consequently, instead of manipulating METTL3, we treated HCC cells with cycloleucine to reduce global RNA methylation. This treatment markedly suppressed *PLK1* mRNA levels without affecting IGF2BP3 protein expression. Collectively, these findings demonstrate that *PLK1* mRNA is subject to m6A modification in HCC, thereby providing a potential mechanistic basis for IGF2BP3-mediated regulation of m6A-modified *PLK1* mRNA.

This study identified that the IGF2BP3-PLK1 axis might act as a promising therapeutic target. However, several limitations require further investigation. First, current conclusions are primarily based on in vitro evidence and need in vivo validation. Xenograft models need to be established to confirm the role of this axis in metabolic reprogramming and to assess PLK1 inhibitors as an intervention. Second, the specific m6A sites on *PLK1* mRNA that mediate IGF2BP3 binding need to be identified via bioinformatics and dual-luciferase assays. Third, the mechanisms by which this axis impairs mitochondrial oxidative phosphorylation and favors glycolysis warrant systematic study. Fourth, given the canonical mitotic function of PLK1 in regulating cell cycle progression and cell proliferation, alterations in proliferation rate or cell cycle distribution may serve as a confounding contributor to the observed metabolic phenotype. This study has not formally excluded this possibility. Further proliferation-independent metabolic assays and cell cycle distribution profiling are required to disentangle the direct metabolic regulatory effect of the IGF2BP3-PLK1 axis from its classical mitotic activity. Fifth, the conventional ROC analysis used in this study does not account for censoring in time-to-event data, and time-dependent ROC methodology was not applied. Additionally, definitive quantification of metabolic flux would require stable isotope-resolved metabolomics, which is beyond the scope of the current study. Nevertheless, our comprehensive bioenergetic profiling provides a compelling functional framework for the observed phenotype.

Together in light of available clinical data on PLK1 inhibitors, our preliminary findings cautiously suggest that combining PLK1 and IGF2BP3 inhibitors may have anti-tumor potential in HCC. This work may offer a preliminary conceptual framework and could provide directional clues for future mechanistic and translational studies on this axis, although these ideas require experimental verification.

## 4. Materials and Methods

### 4.1. Cell Lines and Cell Culture

The human hepatocellular carcinoma cell lines were obtained from the American Type Culture Collection (ATCC, Manassas, VA, USA). SNU387, SNU398, and SNU449 cells were cultured in RPMI-1640 (10% FBS). PLC/PRF/5 cells were maintained in MEM (10% FBS). All cell lines were incubated at 37 °C with 5% CO_2_. Lentiviral particles for *IGF2BP3* and *PLK1* overexpression were purchased from GeneCopoeia (Rockville, MD, USA). *IGF2BP3* knockout was generated using the CRISPR/Cas9 system. *PLK1*-targeting siRNAs were obtained from Hysigen Biosciences (Suzhou, China). The *METTL3* overexpression plasmid and the corresponding negative control were purchased from Wuhan Miaoling Biotechnology Co., Ltd. (Wuhan, China). *METTL3* knockdown was mediated by pLKO.1 puro vectors expressing shRNAs targeting *METTL3*. Transfections were performed using Lipo8000™ transfection reagent (C0533, Beyotime, Shanghai, China). All cell lines were regularly authenticated and tested negative for mycoplasma contamination.

### 4.2. DNA, RNA Extraction, Quantitative PCR, and Quantitative Reverse Transcription RT PCR (qRT-PCR)

Total DNA and RNA were extracted using the DNeasy Blood & Tissue Kit (69506, QIAGEN, Hilden, Germany) and RNAiso Easy (TCH020, Takara, Kusatsu, Japan), respectively. Subsequent cDNA synthesis and qRT-PCR assays were conducted with the First-Strand cDNA Synthesis Kit (QP056, GeneCopoeia, Rockville, MD, USA). QPCR and qRT-PCR assays were both conducted with SYBR Green Real-time PCR Mix (A616690, Sangon, Shanghai, China). Mitochondrial content is represented by the mtDNA/nDNA ratio (*TL1* and *COX1* copy numbers normalized by *VDAC1* copy number). The primers are listed in Table S2.

### 4.3. Western Blot Assay

Reagents and consumables included the BCA assay kit (T9300A, Takara, Kusatsu, Japan) and nitrocellulose membranes (YA1710, Labselect, Hefei, China). Western blot antibodies (dilution, catalog number, manufacturer) were: β-Actin (1:1000, 66009-1-AP, Proteintech, Rosemont, IL, USA), IGF2BP3 (1:1000, 14642-1-AP, Proteintech, Rosemont, IL, USA), PLK1 (1:1000, 37-7000, Thermo Fisher Scientific, Waltham, MA, USA), METTL3 (1:1000, 15073-1-AP, Proteintech, Rosemont, IL, USA), HK2 (1:1000, 66974-1-Ig, Proteintech, Rosemont, IL, USA), NDUFB8 (1:1000, CY8290, Abways, Shanghai, China), SDHB (1:1000, WLR0127, Wanlei, Shenyang, China), UQCRC2 (1:1000, WLR0116, Wanlei, Shenyang, China), MTCO2 (1:1000, S0B1158, Starter, Hangzhou, China), ATP5A1 (1:1000, 14676-1-AP, Proteintech, Rosemont, IL, USA).

### 4.4. Cell Migration and Proliferation Assay

Cell migration, proliferation, and long-term proliferative capacity were evaluated using Transwell inserts, the Cell Counting Kit-8 (CCK-8, GK10001, Glpbio Technology, Montclair, NJ, USA), and colony formation assays, respectively.

### 4.5. Immunohistochemistry (IHC) Assay

Immunohistochemical staining was performed on commercially purchased tissue microarray (HLivH180Su18, HLivH160CS01, Shanghai Outdo Biotech, Shanghai, China). Digital images were captured under a 20× objective lens with consistent exposure settings across all sections. Five random fields per section were acquired for quantitative analysis. IHC staining was evaluated using the ImageJ IHC Profiler plugin (version 1.0). The plugin performs color deconvolution to separate DAB and hematoxylin signals, then classifies DAB intensity into four categories based on pixel intensity thresholds: negative (0), low positive (1+) (score 1), positive (2+) (score 2), and high positive (3+) (score 3). The percentage of cells in each category was automatically calculated. H-score was then derived using the formula: H-score = (% low positive × 1) + (% positive × 2) + (% high positive × 3). Image acquisition and analysis were performed with the operator blinded to sample identity and clinicopathological data.

### 4.6. Luciferase Reporter Assay

PcDNA3-based vectors encoding wildtype and KH domain mutant FLAG-tagged IGF2BP3 (mutational GXXG-GEEG conversion) were kindly provided by Dr Jianjun Chen, Huilin Huang (University of Cincinnati College of Medicine, US). PcDNA3-based vectors encoding RRM domain truncation of FLAG-tagged IGF2BP3 (deleting 2-156aa) were produced by Vertex Biotech (Suzhou, China). Dual-luciferase reporter assays were performed in cells co-transfected with the pMIR-Report-PLK1-3′UTR reporter plasmid, the pRL-TK Renilla luciferase control vector, and WT or MT IGF2BP3 expression plasmids, using Lipo8000™ transfection reagent (C0533, Beyotime, Shanghai, China). Firefly and Renilla luciferase activities were measured using the Dual-Luciferase^®^ Reporter Assay System (E1960, Promega, Madison, WI, USA) according to the manufacturer’s instructions. Firefly luciferase activity was normalized to Renilla luciferase activity.

### 4.7. M6A mRNA Immunoprecipitation (MeRIP)

Total RNA was extracted using TRIzol™ (15596018CN, Invitrogen, Shanghai, China). Subsequent m6A RNA immunoprecipitation (MeRIP) was performed using the EpiQuik™CUT&RUN m6A RNA Enrichment (MeRIP) Kit (P-9018-24, Epigentek Group Inc., Farmingdale, NY, USA) following the manufacturer’s protocol. Briefly, RNA was incubated with an-ti-m6A rabbit polyclonal antibody (an equal amount of non-immune IgG as a negative control) and affinity beads for 90 min at room temperature with rotation. After cleavage with the cleavage enzyme mix and proteinase K digestion, the m6A-containing RNA fragments were purified and eluted. An aliquot of RNA was saved prior to immuno-precipitation as input. The relative m6A enrichment on target transcripts was quantified as percentage of input recovery (% input).

### 4.8. RNA Immunoprecipitation Assay (RIP)

RIP was performed using the Magna RIP™ RNA-Binding Protein Immunoprecipitation Kit (17-700, Merck Millipore, Darmstadt, Germany) according to the manufacturer’s instructions. Briefly, cell lysates were incubated with protein magnetic beads conjugated with an anti-IGF2BP3 antibody (#14642-1-AP, Proteintech, Rosemont, IL, USA) or normal rabbit IgG as a negative control. The immunoprecipitated RNA was purified and subjected to qRT-PCR analysis. The binding of IGF2BP3 to target transcripts was quantified as percentage of input recovery (% input).

### 4.9. Dot Blot Assay

Total RNA was extracted using TRIzol™ reagent (Invitrogen, Carlsbad, CA, USA) and purified using an RNA purification kit (DP412, TIANGEN, Beijing, China). Equal amounts of RNA were denatured and spotted onto Hybond-N+ nylon membranes (GE Healthcare, Chicago, IL, USA). The membranes were UV-crosslinked, blocked with 5% non-fat milk, and incubated with an anti-m6A antibody (#56593S, Cell Signaling Technology, Danvers, MA, USA) overnight at 4 °C. After washing, the membranes were incubated with horseradish peroxidase-conjugated secondary antibody, and signals were visualized using an enhanced chemiluminescence system. Methylene blue staining was performed to verify equal RNA loading.

### 4.10. Mito-tracker and Reactive Oxygen Species Assay

Mitochondria mass/charge were visualized using Mito-tracker Red CMXRos (C1045) or Mito-tracker Green (C1048, Beyotime, Shanghai, China), with nuclei counterstained using Hoechst 33342 (A3472, APExBIO, Houston, TX, USA). Intracellular ROS levels were assessed using the Reactive Oxygen Species Assay Kit (S0034S, Beyotime, Shanghai, China).

### 4.11. Metabolite and Redox State Assays

Levels of glucose-6-phosphate (G6P), lactate, NADP^+^/NADPH ratio, and GSH/GSSG were quantified using the G6P Assay Kit (S0185, Beyotime, Shanghai, China), Lactate Assay Kit (A019-2, Nanjing Jiancheng Bioengineering Institute, Nanjing, China), NADP^+^/NADPH Assay Kit with WST-8 (S0179, Beyotime, Shanghai, China), and GSH/GSSG Assay Kit (S0053, Beyotime, Shanghai, China), respectively.

### 4.12. Seahorse XF Assay

ECAR and OCR were determined using a Seahorse XF Pro Analyzer (Agilent Technologies, Santa Clara, CA, USA). ECAR was measured with the Seahorse XF Glycolysis Rate Assay Kit (cat. no. 103344-100, Agilent Technologies, Santa Clara, CA, USA), and OCR was measured with the Seahorse XF Cell Mito Stress Test Kit (cat. no. 103015-100, Agilent Technologies, Santa Clara, CA, USA). Cells were seeded at a density of 8000 cells per well in XF microplates and cultured overnight prior to the assay. Following the assay, cells in each well were immediately lysed, and total protein concentration per well was quantified using a BCA protein assay. All OCR and ECAR raw values were normalized to the total protein content from the same well. Results are expressed as pmol/min/μg protein for OCR and mpH/min/μg protein for ECAR. The label “Norm. Unit” in the corresponding quantitative bar graphs denotes the “/μg protein” (per μg protein). All experiments were performed with at least 3 independent biological replicates, with a minimum of 3 technical replicates per experimental condition in each assay. Raw data were processed and analyzed using Wave Desktop 2.6 software (Agilent Technologies, Santa Clara, CA, USA). Core functional parameters derived from the assays included basal respiration, maximal respiration, and spare respiratory capacity for OCR, as well as basal glycolysis and compensatory glycolysis for ECAR.

### 4.13. Statistical Analysis

All statistical analyses were performed using R (v4.2.2) and GraphPad Prism (v10.3.0). For comparisons between two independent groups, Student’s *t*-test was used for normally distributed data, and the Mann–Whitney U test was used for non-normally distributed data. Categorical variables were compared using the chi-square test. One-way analysis of variance (ANOVA) followed by Tukey’s HSD post hoc test was used for multiple-group comparisons. The Dunnett test was applied when comparing multiple experimental groups against a single control. Time-gradient experiments (e.g., proliferation curves and mRNA half-life assays) were analyzed using two-way ANOVA. For high-throughput data, multiple comparison corrections were performed using the Benjamini–Hochberg false discovery rate (FDR). Gene set enrichment analysis (GSEA) was performed using permutation tests to evaluate pathway enrichment. Pearson correlation analysis was used to assess linear associations between continuous variables. The mRNA half-life was calculated by nonlinear fitting of a first-order exponential decay model. Survival analysis was conducted with overall survival (OS) as the endpoint. Gene expression was dichotomized at the cohort median, a prespecified strategy adopted to avoid cutoff selection bias. Kaplan–Meier survival curves were generated and compared using the log-rank test. Hazard ratios (HRs) and 95% confidence intervals (CIs) were estimated using univariate and multivariate Cox proportional hazards regression models. The prespecified multivariate model included four established prognostic covariates for HCC: age, sex, grade, and stage. Missing data were handled using complete-case analysis. The proportional hazards (PH) assumption was verified using Schoenfeld residual tests (Figure S3), and all covariates in the multivariate models for *IGF2BP3* and *PLK1* met the PH assumption (all *p* > 0.05). Receiver operating characteristic (ROC) analysis was used to evaluate prognostic performance, with OS as the endpoint and corresponding area under the curve (AUC) values reported. All cell and molecular experiments included at least three independent biological replicates. Statistical analyses were based on the means of biological replicates, while technical replicates were used only to reduce measurement error. Data are presented as mean ± standard deviation (SD). *p* < 0.05 was considered statistically significant (* *p* < 0.05, ** *p* < 0.01, *** *p* < 0.001, **** *p* < 0.0001).

## Data Availability

Publicly available multi-omics and gene expression data used in this study were downloaded from the TCGA Pan-Cancer Analysis of Whole Genomes (PCAWG) project via the ICGC Data Portal (https://dcc.icgc.org/pcawg, accessed on 12 June 2023) and the NCI Genomic Data Commons (GDC) portal (https://portal.gdc.cancer.gov/, accessed on 12 June 2023). The m6A epitranscriptomic microarray dataset generated in this study has been deposited in the Open Archive for Miscellaneous Data (OMIX), National Genomics Data Center, China National Center for Bioinformation/Beijing Institute of Genomics, Chinese Academy of Sciences (https://ngdc.cncb.ac.cn/omix, accessed on 1 October 2026, OMIX ID: OMIX021159) [52,53].

## Supplementary Materials

The following supporting information can be downloaded at: https://www.mdpi.com/article/10.3390/ijms27209245/s1.
