# Supplementary material for: IGF2BP3 Orchestrates Metabolic Reprogramming via Stabilizing m6A-Modified PLK1 mRNA to Promote Hepatocellular Carcinoma Progression
Source: Int J Mol Sci. 2026 Oct 18;27(20):9245. doi: 10.3390/ijms27209245 (PMC13704930; doi:10.3390/ijms27209245)
Supplement: Supplementary file 1 [file ijms-27-09245-s001.zip › ijms-4519752-supplementary.pdf]

Supplementary Materials for

IGF2BP3 Orchestrates Metabolic Reprogramming via Stabilizing m6A-Modified PLK1 mRNA to Promote Hepatocellular Carcinoma Progression

* Correspondence to:

Dr. Xiaobo Cui:

Department of Medical Genetics, School of Basic Medical Sciences,

Harbin Medical University, No. 157, Baojian Road, Nangang District,

Harbin 150081, China.

Email: cuixiaobo@hrbmu.edu.cn

Dr. Cize Gao

Department of Medical Genetics, School of Basic Medical Sciences,

Harbin Medical University, No. 157, Baojian Road, Nangang District,

Harbin 150081, China.

Email: gaocize@hrbmu.edu.cn

Supplementary materials and methods

Data acquisition and preprocessing

Public datasets

Bulk transcriptomic and clinical data for the TCGA-LIHC cohort (374 HCC tumors, 50 adjacent non-tumor tissues; GRCh38/hg38 genome assembly) were obtained from the Genomic Data Commons Data Portal (https://portal.gdc.cancer.gov/). Raw HTSeq read counts were normalized to transcripts per million (TPM) for expression level comparison, voom-transformed, and analyzed for expression-level comparisons using the *limma* package (v3.62.2).

For transcriptomic validation, nine independent GEO datasets (https://www.ncbi.nlm.nih.gov/geo/) were used for transcriptomic validation, covering Affymetrix microarrays, Illumina bead arrays, and RNA-seq platforms (accession numbers: GSE138178, GSE6764, GSE17548, GSE25097, GSE136247, GSE112790, GSE36376, GSE45436, GSE64041). For Affymetrix array datasets, raw CEL files were processed with robust multi-array average (RMA) background correction followed by quantile normalization via the *affy* package (v1.82.0). For Illumina array and RNA-seq datasets, processed expression matrices were downloaded directly, log_2-transformed, and annotated using official platform annotation files; probes mapping to multiple gene symbols were collapsed to the median expression value per gene.

Additional cross-cohort expression validation was performed using the TNMplot V2 web server (https://tnmplot.com/analysis/). Normalized expression values were retrieved with default settings: Mann-Whitney U test for group comparison and default normalization across all included data sources (GEO, GTEx, TCGA, TARGET).

CPTAC-LIHC proteomic data (159 primary HCC tumors, 110 adjacent non-tumor

tissues; TMT 10-plex labeling, GRCh38 annotation) were downloaded from the CPTAC Data Portal (https://pdc.cancer.gov/pdc/browse/). Missing values were imputed using the k-nearest neighbors algorithm, and protein identifiers were mapped to official human gene symbols.

Single-cell RNA-seq data of HCC (dataset ID: *LiHC_GSE125449_aPDL1aCTLA4*) were retrieved from the Tumor Immune Single Cell Hub 2 (TISCH2, http://tisch.compbio.cn/home/). Standard quality control, LogNormalize normalization (scale factor = 10,000), UMAP dimensionality reduction, and cell type annotation were pre-performed by the database as its standard processing pipeline. Malignant cell identity was pre-defined by inferCNV analysis in the database.

In-house m^{6}A epitranscriptomic microarray

m^{6}A-modified mRNA profiles were generated in-house using human m^{6}A epitranscriptomic microarrays (Arraystar, Rockville, MD, USA) with five paired HCC and adjacent non-tumor liver tissues. Total RNA was isolated from each sample, and m^{6}A-modified mRNA was enriched via anti-m^{6}A immunoprecipitation followed by hybridization to the microarray. Raw microarray data were background-corrected and quantile-normalized using the *limma* package (v3.62.2). Differentially m^{6}A-modified transcripts between tumor and non-tumor tissues were defined by a cutoff of $|\log_2 \text{fold change}| \geq 1$ and $p < 0.05$.

Identification of differently expressed genes (DEGs) in LIHC

Differential expression between groups in both RNA-seq and m6A-epitranscriptome microarray datasets was performed with the *limma* package (v3.62.2), GEO2R was used for differential expression analysis of GEO datasets and p value < 0.05 were considered differentially expressed for all

cohorts.

Survival Analysis

Overall survival (OS) was defined as the time from diagnosis to all-cause death. OS information and gene expression matrices were integrated using the survival package (v3.8-6). Missing clinical covariates were handled using complete-case analysis; samples with missing data in any included covariate were excluded from multivariate analyses. The final TCGA-LIHC discovery cohort included 344 patients with 114 death events for multivariate survival analysis. Patients were stratified into high- and low-expression groups based on the cohort median expression value of each gene. Kaplan-Meier survival curves were generated and compared by the log-rank test. Univariate and multivariate Cox proportional hazards regression models were fitted to evaluate the independent prognostic value of each gene. The prespecified multivariate model included four well-established prognostic covariates for HCC: age at diagnosis, sex, grade, and stage. The proportional hazards (PH) assumption was verified using Schoenfeld residual tests. The global Schoenfeld tests confirmed that both the *IGF2BP3* (p = 0.3171) and *PLK1* (p = 0.6675) multivariate models satisfied the PH assumption, with all individual covariates showing no significant time-dependent deviation (all $p > 0.05$). Detailed residual plots are provided in Figure S3. Receiver operating characteristic (ROC) analysis was performed using the *pROC* package (v1.18.5) to assess the prognostic performance of gene expression, with OS as the endpoint. AUC values and their 95% confidence intervals (CIs) were calculated, and $p < 0.05$ was considered statistically significant. Additionally, the GEPIA2 web server (http://gepia2.cancer-pku.cn/) was used to perform survival analysis for HK2 in LIHC. Kaplan-Meier survival curves for OS were generated based on the TCGA-LIHC dataset, using median expression as the group cutoff. Log-rank

tests and hazard ratios (HRs) with 95% CIs were calculated to evaluate the association between HK2 expression and patient survival.

Functional enrichment analysis

Functional enrichment of DEGs was performed with Metascape: https://metascape.org. Gene Ontology (GO) term enrichment was evaluated using Fisher's exact test with Benjamini-Hochberg FDR correction. FDR < 0.05 was considered significant.

Pearson Correlation Analysis

Pearson correlation analysis was performed using the cor.test() function to obtain the correlation coefficient (*r*) and *p* value; *r* ranged from -1 to 1, and $p < 0.05$ was considered statistically significant. Additionally, the GEPIA2 web server was used to evaluate the correlations among *IGF2BP3, PLK1,* and *HK2* based on the TCGA-LIHC dataset.

Pan-Cancer Analysis

IGF2BP3 and *PLK1* mRNA levels in cancerous versus matched non-cancerous tissues across multiple cancer types were compared using the Tumor Immune Estimation Resource 2.0 (TIMER 2.0): http://timer.cistrome.org/. $\log_2$ |fold change| was computed, and Benjamini-Hochberg FDR correction was applied. FDR < 0.05 considered statistically significant.

Single-cell sequencing analysis

The above TISCH2 single-cell dataset was used for downstream analysis. Malignant cells were further validated by hepatocellular-carcinoma-specific

epithelial marker genes (EPCAM, KRT19, AFP, ASGR1, GPC3), defined as cells with mean marker expression in the top 25th percentile across all cells. Cells were then stratified into high and low expression groups by the median expression of IGF2BP3 and PLK1, and the proportion of malignant cells between groups was compared using Chi-square test. $p < 0.05$ was considered statistically significant.

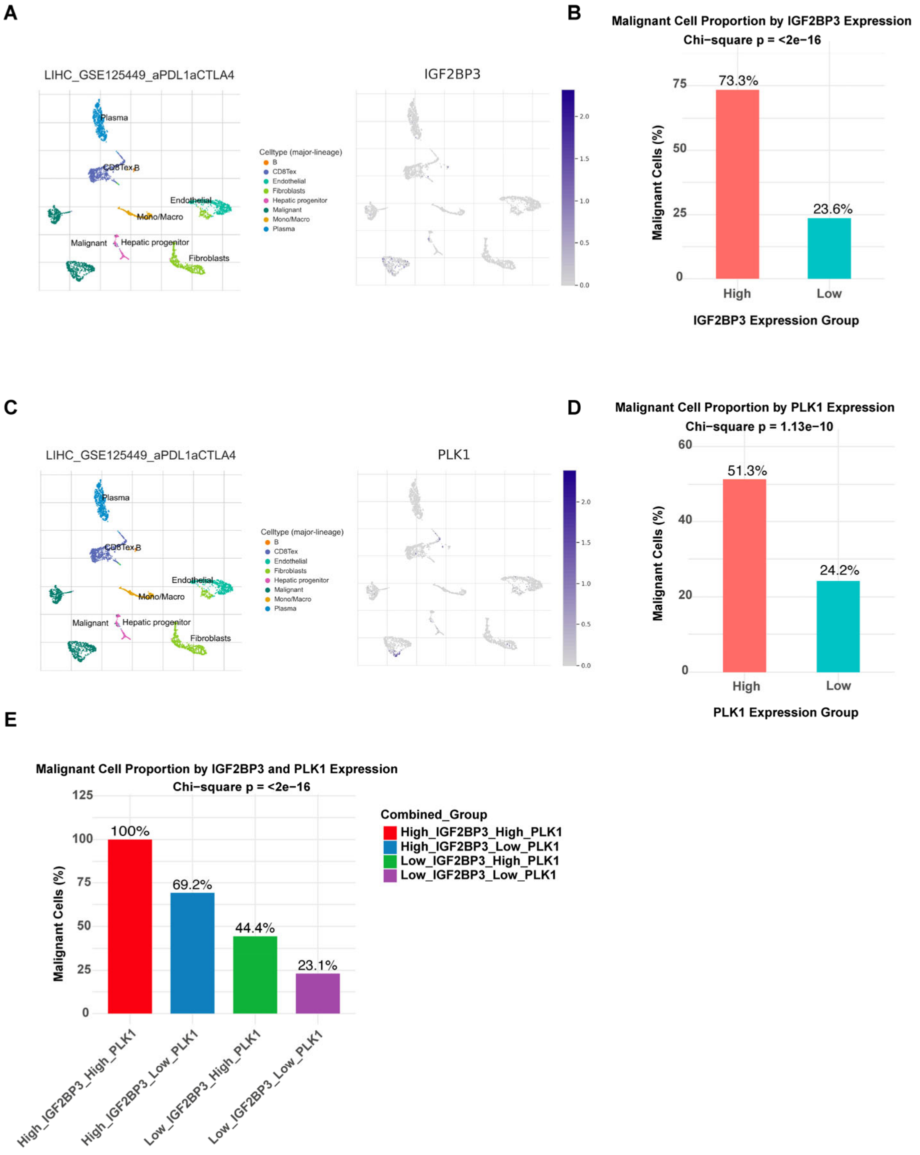

Figure S1. Single-cell analysis of *IGF2BP3* and *PLK1* expression in HCC and their association with malignant cell fraction. (A, C) UMAP projections (TISCH2) showing *IGF2BP3* and *PLK1* expression levels across cell types in HCC microenvironment. (B, D) Comparison of malignant cell proportions between high- and low-expression groups for *IGF2BP3* and *PLK1*, respectively. (E) Malignant cell proportions stratified by combined *IGF2BP3*/*PLK1* expression. Malignant cells were identified via inferCNV, and groups were defined by median expression.

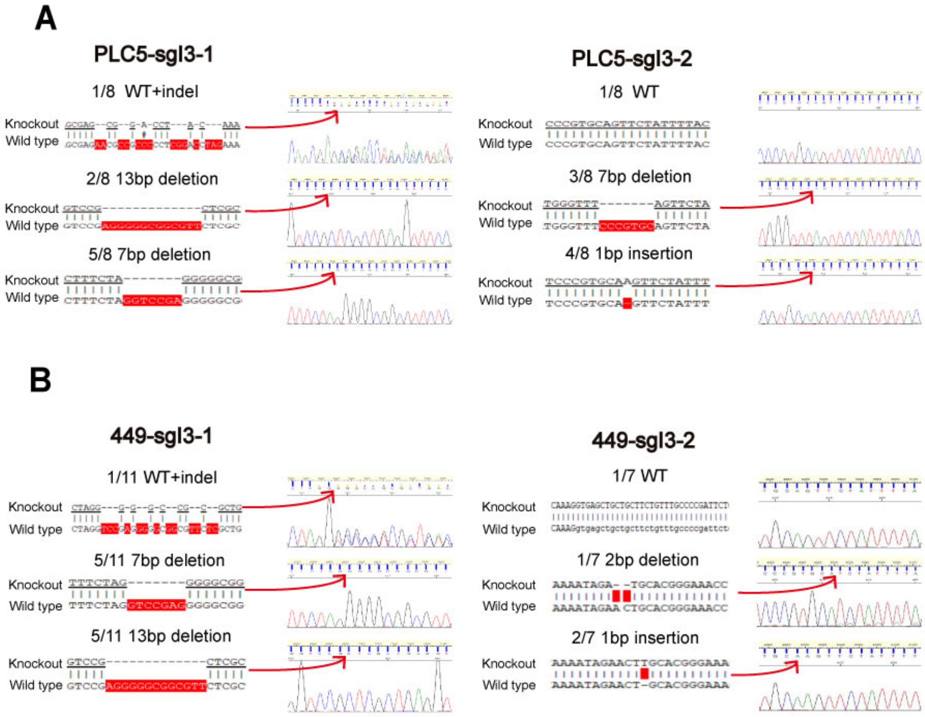

Figure S2. Sanger sequencing validation of CRISPR-Cas9-mediated *IGF2BP3* knockout in PLC/PRF/5 and SNU449 clones. (A) PLC5-sgI3-1 and sgI3-2. (B) 449-sgI3-1 and sgI3-2. For each clone, left panels show WT and KO genomic sequences with mutation details; right panels display sequencing chromatograms (red arrows indicate mutation sites).

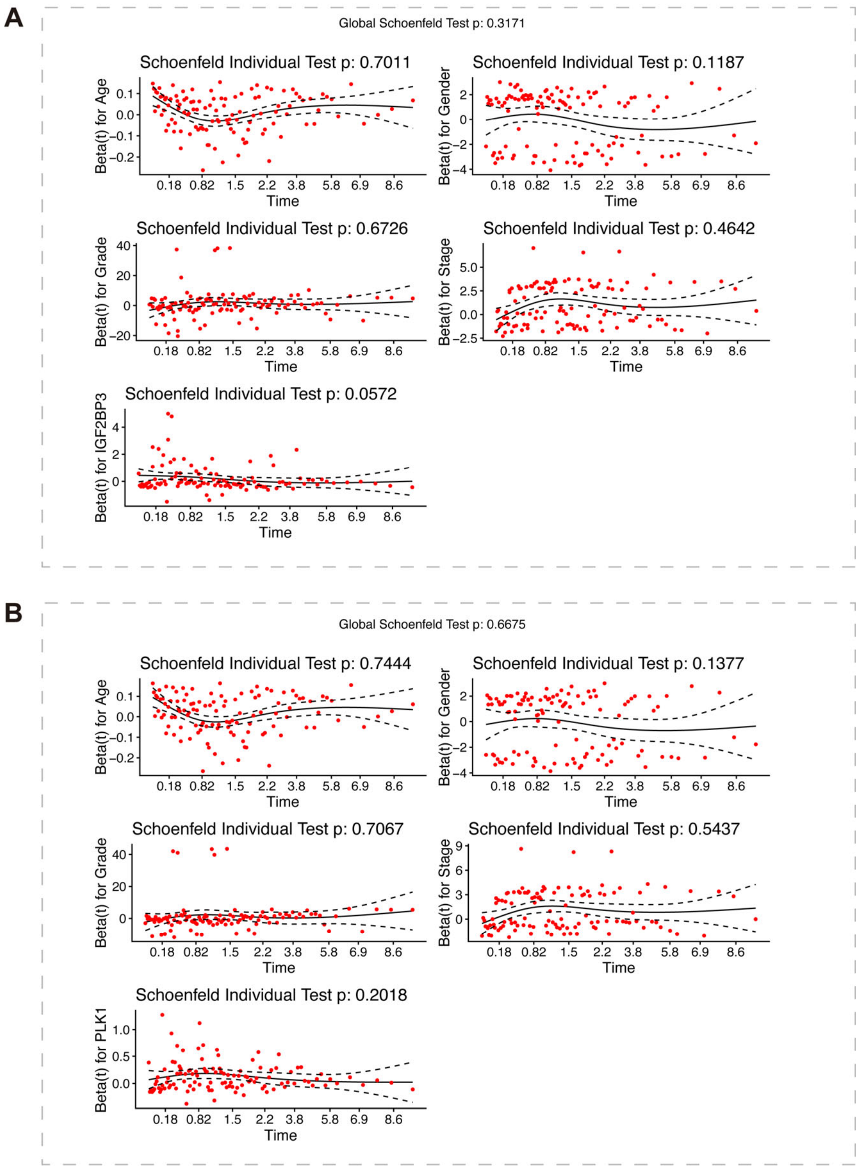

Figure S3. Proportional hazards assumption verification by Schoenfeld residual tests. (A) Multivariate Cox model for *IGF2BP3* (Fig. 1H), adjusted for age, sex, grade and stage. The global Schoenfeld test confirmed overall compliance with the proportional hazards assumption (p = 0.3171), and individual tests for all covariates showed no significant time-dependent deviation (all $p > 0.05$). (B) Multivariate Cox model for *PLK1* (Fig. 3I), adjusted for age, sex, grade and stage. The global Schoenfeld test confirmed overall compliance with the proportional hazards assumption (p = 0.6675), and individual tests for all covariates showed no significant time-dependent deviation (all $p > 0.05$).

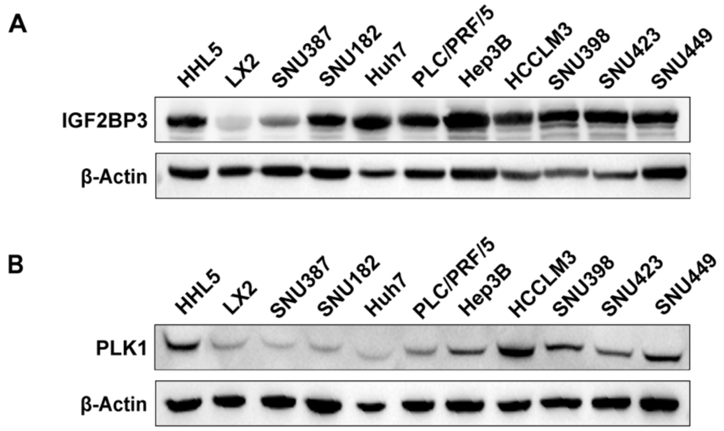

Figure S4. Baseline protein expression of IGF2BP3 and PLK1 across human normal liver cell lines and HCC cell lines. (A) Endogenous IGF2BP3 protein levels were examined by Western blot, including two non-malignant lines (HHL5, normal human hepatocyte line; LX2, human hepatic stellate cell line) and nine HCC cell lines (SNU387, SNU182, Huh7, PLC/PRF/5, Hep3B, HCCLM3, SNU398, SNU423, and SNU449). (B) Endogenous PLK1 protein levels in the same panel of cell lines. β-Actin was used as a loading control in both panels. All experiments were independently repeated at least three times.

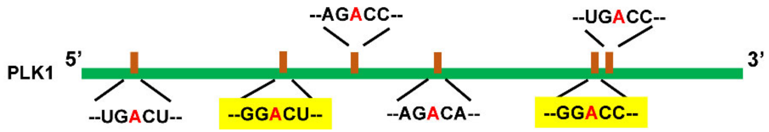

Figure S5. Prediction of putative m6A modification sites in *PLK1* transcript by SRAMP.

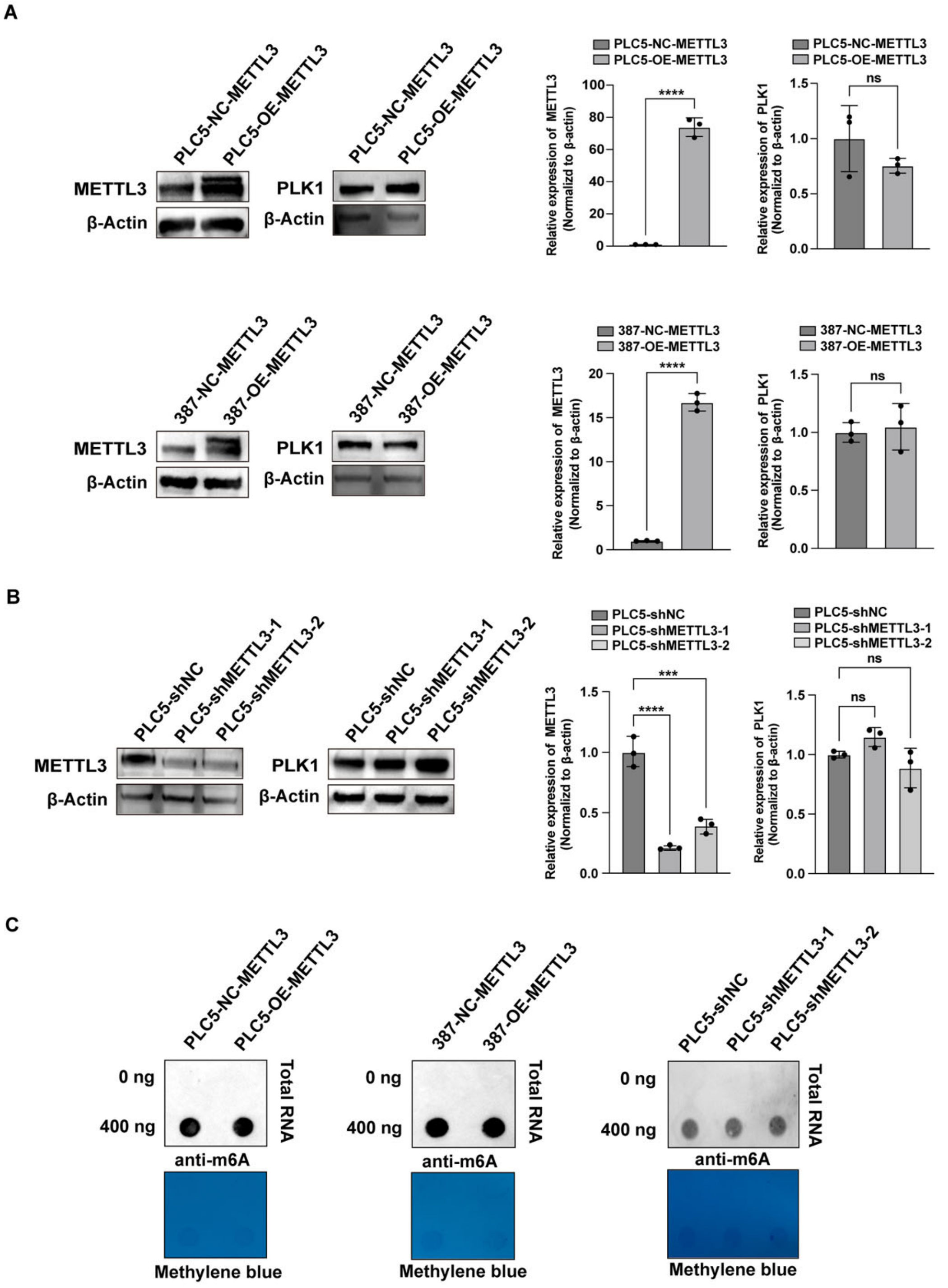

Figure S6. Effects of *METTL3* overexpression and knockdown on *PLK1* expression and global m6A levels. (A) Western blot analysis of METTL3 and PLK1 protein levels (left) and qRT-PCR analysis of *METTL3* and *PLK1* mRNA levels (right) in PLC/PRF/5 and SNU387 cells with *METTL3* overexpression. β-Actin served as the internal reference. (B) Western blot analysis of METTL3 and PLK1 protein levels (left) and qRT-PCR

analysis of *METTL3* and *PLK1* mRNA levels (right) in PLC/PRF/5 cells with *METTL3* knockdown. β-Actin served as the internal reference. (C) Dot blot assay detecting global m6A levels of total RNA in each group. Methylene blue staining was performed to verify equal sample loading. Data are presented as mean ± SD from three independent replicates. (* $p < 0.05$, ** $p < 0.01$, *** $p < 0.001$, **** $p < 0.0001$. ns, not significant.).

Table S1. Clinicopathological characteristics of the two HCC TMA cohorts.

Characteristic	Cohort 1 (n = 52)	Cohort 2 (n = 88)
**Histological grade, n (%)**		
Low grade (I /I–II/ II)	23 (44.2)	63 (71.6)
High grade (II–III/III/IV)	29 (55.8)	25 (28.4)
**AJCC stage, n (%)**		
Early stage (I–II)	24/42 (57.1) *	50/81 (61.7)
Advanced stage (III–IV)	18/42 (42.9) *	31/81 (38.3)
Tumor size, cm, mean ± SD	8.37 ± 4.33	7.67 ± 5.37
**Follow-up**		
Median follow-up, months	Not available	33 (range: 24–44)
Death events, n (%)	Not available	44 (50.0)
Median overall survival, months	Not available	26

* Ten cases in Cohort 1 and seven cases in Cohort 2 had incomplete AJCC staging information and were excluded from stage-stratified analyses.

Histological grades were dichotomized into low grade (grade I, I–II, and II) and high grade (grade II–III, III, and III–IV), consistent with the grouping in the main text figures and the covariate stratification used in multivariable Cox regression models.

Tumor size represents the largest tumor dimension in centimeters.

Median follow-up was calculated among censored (alive) patients in Cohort 2. Age and sex data were not available in either TMA clinical dataset.

Abbreviations: HCC, hepatocellular carcinoma; TMA, tissue microarray; AJCC, American Joint Committee on Cancer; SD, standard deviation.

Table S2. Sequences of sgRNAs targeting IGF2BP3, siRNAs targeting PLK1, shRNAs targerting METTL3 and qPCR primers.

sg-*IGF2BP3*-1	5'- GATACTTTCTAGGTCCGAGGGGG -3'
sg-*IGF2BP3*-2	5'- GCAGGTAAAATAGAACTGCACGG -3'
si-*PLK1*-1	5'- GTTCTTTACTTCTGGCTATAT -3'
si-*PLK1*-2	5'- CAACGGCAGCGTGCAGATCAA -3'
sh-*METTL3*-1	GCCAAGGAACAAUCCAUUGUUTT
sh-*METTL3*-2	GCUGCACUUCAGACGAAUUAUTT
Human *β-actin*-F	GTGGCCGAGGACTTTGATTG
Human *β-actin*-R	CCTGTAACAACGCATCTCATATT
Human *IGF2BP3*-F	GGATGCGTTTGGGTTGTAGC
Human *IGF2BP3*-R	TAAAGACCGGAACACCACGC
Human *PLK1*-F	CACCAGCACGTCGTAGGATTC
Human *PLK1*-R	CCGTAGGTAGTATCGGGCCTC
Human *METTL3*-F	AGATGGGGTAGAAAGCCTCCT
Human *METTL3*-R	TGGTCAGCATAGGTTACAAGAGT
Human *CoxI*-F	TTCGCCGACCGTTGACTATTCTCT
Human *CoxI*-R	AAGATTATTACAAATGCATGGGC
Human *TL1*-F	CACCCAAGAACAGGGTTTGT
Human *TL1*-R	TGGCCATGGGTATGTTGTTA
Human *VDAC1*-F	TCTGGTGGTGCATCATGGAG
Human *VDAC1*-R	CGTGGGTGCTGGTCCTTATT
